# Web-Based Guided Self-Help vs Treatment as Usual for Binge-Eating Disorder: A Randomized Clinical Trial

**DOI:** 10.1001/jamanetworkopen.2025.36644

**Published:** 2025-10-10

**Authors:** Ella van Beers, Bernou Melisse, Petra Poelstra, Nick Lommerse, Margo de Jonge, Marleen de Waal, Jaap Peen, Edwin de Beurs, Elske van den Berg

**Affiliations:** 1 Novarum Centre for Eating Disorders and Obesity, Amstelveen, the Netherlands; 2 Department of Clinical Psychology, Leiden University, Leiden, the Netherlands; 3 American Center for Psychiatry and Neurology, Al Manhal, Abu Dhabi, United Arab Emirates; 4 Co-Eur, Utrecht, the Netherlands; 5 Arkin Mental Health Institute, Amsterdam, the Netherlands

## Abstract

**Importance:**

Preliminary findings suggest that web-based guided self-help, which is easily scalable and shorter in duration, may be an effective treatment for binge-eating disorder, yet it is rarely offered or only used as a first-line treatment in a stepped-care approach. Despite its benefits and cost savings, few studies have directly compared it with treatment as usual. Evaluating a psychological treatment that requires minimal therapist involvement is crucial for reducing costs, improving accessibility, and promoting patient agency in recovery.

**Objective:**

To assess whether web-based guided self-help is noninferior to treatment as usual for binge-eating disorder.

**Design, Setting, and Participants:**

This 2-arm noninferiority randomized clinical trial of adults with binge-eating disorder was conducted between November 2021 and October 2024 at a specialized center for eating disorders in the Netherlands.

**Interventions:**

Participants received either 12 weeks of web-based guided self-help or 20 weeks of comprehensive treatment as usual.

**Main Outcome and Measures:**

The primary outcome was the comparative difference in the number of objective binge-eating episodes at the end of treatment and at 20 weeks after treatment. Secondary measures included full remission, dropout, therapeutic alliance, body dissatisfaction, clinical impairment, and body mass index. To examine differences in outcomes between groups, linear mixed model analyses with restricted maximum likelihood estimation, multilevel negative binomial regression, and multilevel binary logistic regression were used.

**Results:**

Among a referred sample of 207 adults with binge-eating disorder, 187 participants (160 females [85.6%]; mean [SD] age, 38.1 [0.9] years) were randomized to web-based guided self-help (n = 93) or treatment as usual (n = 94). With a margin of 1 additional binge-eating episode, a contrast estimate noninferiority was found between groups at the end of treatment (−0.82 [95% CI, −1.86 to 0.21]; *P* = .12) and at 20 weeks after treatment (−0.58 [95% CI, −1.41 to 0.26]; *P* = .17). At both assessments, a difference in objective binge-eating episodes was found in favor of web-based guided self-help (end of treatment: 3.24 [95% CI, 2.42-4.33] vs 4.06 [3.17-5.20]; 20 weeks after treatment: 1.27 [95% CI, 0.81-1.97] vs 1.84 [95% CI, 1.24-2.73]).

**Conclusions and Relevance:**

In this randomized clinical trial, web-based guided self-help was noninferior to treatment as usual in reducing binge eating, despite its shorter duration and lower level of therapist involvement. These results suggest more evidence for the effectiveness of web-based mental health care, which is an important finding amid global challenges with mental health staff shortages, long waiting lists, and high treatment costs.

**Trial Registration:**

Central Committee on Research Involving Human Subjects Identifier: NL76368.100.21

## Key Points

**Question:**

Is web-based guided self-help noninferior to treatment as usual for adults with binge-eating disorder?

**Findings:**

In this randomized clinical trial of 187 adults with binge-eating disorder, web-based guided self-help was noninferior to treatment as usual in reducing binge-eating episodes at both the end of treatment and at 20 weeks after treatment.

**Meaning:**

The findings of this study suggest that web-based guided self-help is an effective standalone treatment for adults with binge-eating disorder and may be beneficial for challenges in the global mental health field.

## Teaser

This randomized clinical trial of adults with binge-eating disorder assesses whether web-based guided self-help is noninferior to treatment as usual in reducing binge-eating episodes.

## Introduction

Binge-eating disorder (BED) is the most prevalent eating disorder, characterized by recurrent binge-eating episodes and marked distress around binge eating.^1,2^ Unlike other eating disorders, those with BED do not use compensatory behaviors such as fasting or purging.^1^ BED affects approximately 1.5% of women and 0.3% of men worldwide, with a lifetime prevalence of 0.6% to 1.8% for women and 0.3% to 0.7% for men.^3^ BED is associated with medical complications, excessive weight gain, and psychosocial impairment.^4,5,6^

Guidelines recommend specialized individual treatment for BED using a cognitive-behavioral framework, such as cognitive behavior therapy-enhanced (CBT-E).^7^ However, there is little consensus across guidelines worldwide on treatment intensity. In the United Kingdom and Australia, guided self-help is recommended as a first line of treatment and stand-alone therapy,^8,9^ whereas in the Netherlands, Dutch guidelines recommend individual CBT-based treatment as a first line of care.^10^ These contrasting recommendations may be attributed to the lack of conclusive evidence on the effectiveness of guided self-help compared with treatment-as-usual CBT-E. The National Institute for Health and Care Excellence has highlighted the importance of researching the clinical effectiveness of CBT-based guided self-help for adults with BED.^9^

Recommended treatments are successful in achieving full remission from binge eating in approximately 50% of individuals seeking treatment.^11,12,13^ A 2019 meta-analysis, including 81 randomized clinical trials with 7515 individuals, found that the posttreatment abstinence from a binge-eating rate was 53% (95% CI, 45%-61%) greater for in-person psychological treatment compared with an inactive control group.^13^ For self-help programs, an abstinence rate of 46% (95% CI, 33%-59%) over 1 year was found. Although dropout was lower in the in-person psychological treatment group compared with self-help, a lack of dropout heterogeneity among included studies was found (*I*^2^ = 0%).^13^ When comparing treatment outcomes of 3 randomized clinical trials on guided self-help programs with standard treatment, no short-term or long-term differences have been found.^12,13,14,15,16^

Two randomized clinical trials^15,16^ have evaluated a guided self-help program by Fairburn et al^17,18^ but lacked a CBT-E comparison group.^7^ Wilson et al^15^ found no difference in binge-eating abstinence when comparing 19 therapist-led sessions with 10 shorter guided CBT self-help sessions^17^ at the end of treatment and at the 2-year follow-up, although the guided self-help group had more dropout.^15^ Similarly, Peterson et al^16^ found no difference in the number of binge-eating episodes or abstinence from binge eating between 21 therapist-led sessions and 10 guided self-help CBT-E sessions at the end of treatment and at the 6-month follow-up. Lastly, de Zwaan et al^14^ found that 2 90-minute sessions followed by 11 email-based guided self-help sessions were inferior to in-person treatment on reducing days with binge-eating episodes at the end of treatment and at the 6-month follow-up. Interestingly, no difference was found after 1.5 years, and there were no differences in dropout between groups.^14^

Evaluating the effectiveness of guided self-help interventions in psychiatry is critical given the potential advantages of alleviating mental health staff shortages, shortening waiting lists, and lowering treatment costs.^19,20,21^ A systematic review and meta-analysis of 21 studies with 810 participants compared the effect of guided self-help with in-person treatment for depression and anxiety and found no significant differences in treatment effect at the end of treatment or throughout a 1-year follow-up period, and no differences in treatment dropout were found.^22^ Similarly, a systematic review and meta-analysis comparing guided self-help with in-person individual, group, and unguided self-help treatments for panic disorder found no difference in treatment effect between guided self-help and in-person treatments.^23^ Rather, the study found that guided self-help was more effective compared with unguided self-help, with no differences in dropout.^23^

This study aimed to examine whether web-based guided self-help is noninferior to treatment as usual for adults with BED. The design was developed based on the established efficacy and cost-effectiveness of web-based guided self-help^24,25^ and prior work suggesting that standard treatment may be unnecessarily extensive to achieve optimal outcomes for those with BED.^26^

## Methods

This noninferiority randomized clinical trial was conducted between November 2021 and October 2024, covarying for baseline measures. A blinded, 1:1 block randomization with stratification for BMI^27,28,29^ above or below 35 (calculated as weight in kilograms divided by height in meters squared) was performed using a 4-, 6-, and 8-block design in an electronic data capture system (Castor EDC; Castor).^30^ Participants were stratified by BMI, given prior work that suggests BMI may predict treatment response for those with BED.^27,28,29^ Participants waited 4 weeks after randomization to start treatment. An overview of the study method and outcomes is provided in the trial protocol (Supplement 1). Patients and caregivers were involved in the study design, execution, and reporting. Written informed consent was obtained from all participants, and the trial was approved by the Medical Research Ethics Committees United. This study followed the Consolidated Standards of Reporting Trials (CONSORT) reporting guideline.

### Participants

Participants were referred to a specialized eating disorder treatment center in Amsterdam, the Netherlands. Eligibility criteria included a *Diagnostic and Statistical Manual of Mental Disorders* (Fifth Edition) (*DSM-5*) diagnosis of BED or otherwise-specified feeding or eating disorder–BED,^1^ being 18 years or older, proficiency in Dutch, having internet access, and having a BMI over 19.5. Exclusion criteria were acute psychosis, suicidality, or major depressive disorder necessitating stabilization before eating disorder treatment, assessed using the *Structured Clinical Interview for DSM-5*^31^; pregnancy upon study entry; using medications that might influence eating; and receiving eating disorder treatment in the previous 6 months.

### Assessments

Assessments were at baseline, end of treatment, 20 weeks after treatment, and 40 weeks after treatment. In guided self-help, the end of treatment was 12 weeks after the start of treatment, while in the treatment-as-usual group, the end of treatment was 20 weeks after the start of treatment. All assessments used the validated Dutch version of each interview or questionnaire. Adverse events were systematically monitored.

### Interventions

#### Web-Based Guided Self-Help

Web-based guided self-help is a 12-week program based on *Overcoming Binge Eating: The Proven Program to Learn Why You Binge and How You Can Stop* (Second Edition) by Christopher G. Fairburn.^18^ The Dutch-translated version^32^ was used and adapted onto a secure online platform accessible to participants and therapists. van den Berg et al^33^ presents further details on the program. Both programs aim to establish a regular eating pattern, develop alternatives to binge eating, support problem-solving skills, address overevaluation of shape and weight and strict dieting, and promote relapse prevention. Unlike treatment as usual, mood intolerance was not addressed in web-based guided self-help. During weekly, preplanned 20-minute videoconferencing sessions, therapists monitored progress and provided scripted feedback to encourage program adherence and support problem-solving within the program. In addition, participants planned twice-weekly self-review sessions to review progress and to plan the upcoming days.

#### Treatment as Usual

Treatment as usual was the individual focused version of CBT-E,^7^ the recommended treatment for adults with BED in the Netherlands. CBT-E treatment consisted of 20 50-minute videoconferencing sessions over 20 weeks. While treatment would typically be in person, sessions were online due to the COVID-19 pandemic. Prior work suggests that clinical outcomes of videoconferencing are noninferior to in-person treatments in psychiatry.^34,35^

#### Therapists and Assessors

Specialized (predominantly master degree-level) therapists were trained on CBT-E by the Centre for Research on Eating Disorders at Oxford and were trained on the web-based guided self-help program by B.M. All therapists provided treatments across both groups. Assessments were conducted by master degree–level psychologists, who were blinded to the allocation group and supervised by a senior psychologist. Weekly 45-minute supervision sessions were held for therapists. Therapists self-rated their protocol adherence on a Likert scale from 1 (minimal) to 5 (excellent) after each session.

### Measures

The primary outcome was the number of objective binge-eating episodes at the end of treatment and at 20 weeks after treatment. Binge-eating episodes were measured in the previous 28 days using the Eating Disorder Examination (EDE),^36^ a semistructured interview examining the frequency and severity of eating disorder symptoms, in which scores range from 0 to 6, with higher scores indicating more severe eating disorder pathology.^36,37^ The noninferiority margin was set to 1 additional binge-eating episode. The primary outcome was revised from the trial protocol, which proposed examining full remission to provide a more sensitive measure of change and to support comparability with existing literature.

### Secondary Outcomes

This study also measured secondary outcomes. These included the number of binge-free days, the number of binge-eating episodes at 40 weeks after treatment, eating disorder pathology, full remission, dropout, therapeutic alliance, body dissatisfaction, clinical impairment, and BMI. Details on the measurement, assessment time points, and psychometric properties of the secondary outcomes are presented in eTable 1 in Supplement 2.

### Statistical Analysis

#### Sample Size

With a margin of 1 additional binge-eating episode (Δ = 1, α = .05, β = 0.8, and SD = 2.4)^14^ in the previous 28 days at the end of treatment, 144 participants (72 per arm) were needed to establish noninferiority. With 20% expected attrition, 180 participants were to be recruited.

#### Analyses

Primary analyses used an intention-to-treat approach. Baseline characteristics were compared using χ^2^, *t*, and Mann-Whitney tests. Multiple imputation (50 imputation sets) was performed using mice in R, version 3.17.0 (R Project for Statistical Computing).^38^ Both groups were imputed separately. To examine differences in outcomes between groups, linear mixed model analyses with restricted maximum likelihood estimation (continuous normally distributed measures), multilevel negative binomial regression (count variables), and multilevel binary logistic regression (dichotomous variables) were used. Additional details are described in the eMethods in Supplement 2. Estimated marginal means were derived from the multilevel models for all measures and were compared 2-sided between groups. Noninferiority was concluded from the 95% CIs of the mean differences between groups from that test. A per-protocol analysis (ie, exclusion of all participants who dropped out) was performed as a sensitivity analysis. Between-group effect sizes (Cohens *d*) based on pooled SDs with CIs were derived from the linear mixed model analyses.^39^ Due to only the self-reported EDE Questionnaire (EDE-Q), in which scores range from 0 to 6, with higher scores indicating more severe eating disorder pathology, being used at the 40-week posttreatment assessment, the correlation between binge-eating episodes on the EDE and the EDE-Q was analyzed across all earlier assessments. Significance levels were set to a 2-sided *P* < .05. Aside from the imputation performed in R, all other statistical analyses were performed using SPSS, version 29.0 (IBM Corp).^40^

## Results

### Participants

Participant enrollment, dropout, and attrition are presented in the Figure. Of 207 adults with BED or otherwise-specified feeding or eating disorder–BED assessed for study eligibility, 8 (3.9%) refused to participate in the trial, and 187 participants (160 females [85.6%] and 27 males [14.4%]; mean [SD] age, 38.1 [0.9] years; range, 19.0-63.0 years) were randomized to receive web-based guided self-help (n = 93) or treatment as usual (n = 94). Randomization exceeded the target of 180 due to an influx in intakes at the end of the randomization period, after which recruitment ended. Participants had a mean (SD) BMI of 34.2 (0.4 [range, 20.0-43.2]) and reported a mean (SD) eating disorder duration of 22.0 (13.8) years. Among the participants, 159 (85.0%) had not previously received eating disorder treatment. Most participants (167 [89.3%] were born in the Netherlands; however, a baseline difference was observed in the country of birth, with 5 of 93 participants (5.4%) in the web-based guided self-help group born outside of the Netherlands vs 15 of 94 (16.0%) in the treatment-as-usual group. Thus, country of birth was added as a covariate in subsequent analyses (χ^2^_1_ = 5.48; *P* = .02). Participant characteristics are provided in Table 1.

**Figure.**
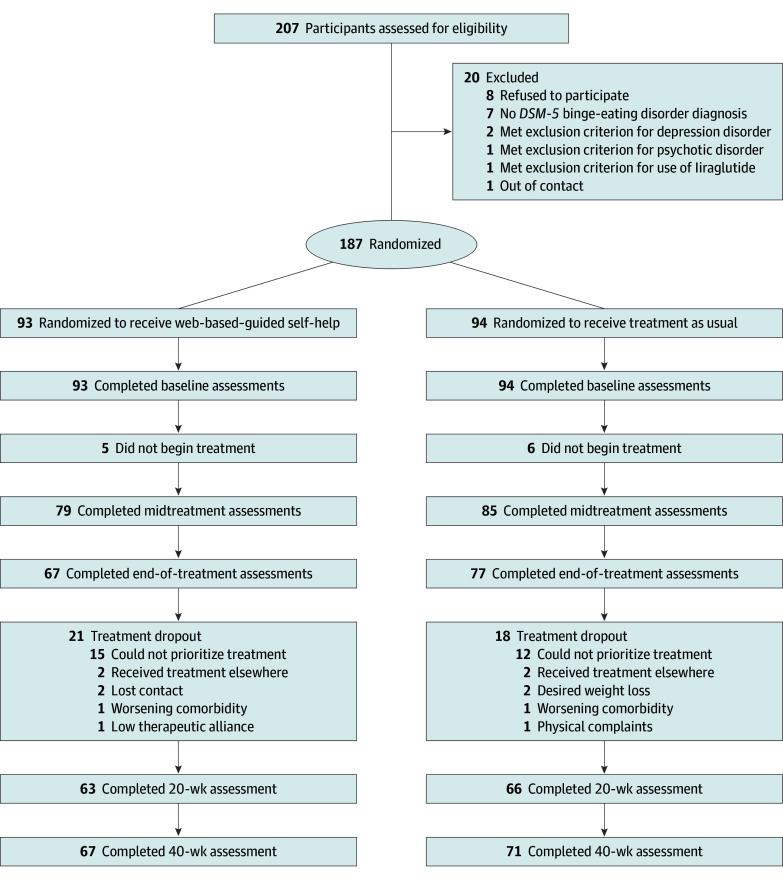
Participant Flow Diagram *DSM-5* indicates *Diagnostic and Statistical Manual of Mental Disorders* (Fifth Edition).

**Table 1.** Baseline Participant Characteristics

Characteristic	Participant group, No. (%)	
	Web-based–guided self-help (n = 93)	Treatment as usual (n = 94)
Age, mean (SD) [range], y	37.4 (11.8) [19.0-63.0]	38.5 (12.5) [19.0-68.0]
BMI, mean (SD) [range]	33.9 (4.9) [20.0-43.1]	34.6 (4.6) [24.3-43.2]
Sex assigned at birth		
Female	80 (86.0)	80 (85.1)
Male	13 (14.0)	14 (14.9)
*DSM-5* diagnosis		
BED	79 (84.9)	79 (84.0)
Otherwise-specified feeding or eating disorder–BED	14 (15.1)	15 (16.0)
Country of birth		
Netherlands	88 (94.6)	79 (84.0)
Other	5 (5.4)	15 (16.0)
Educational level		
Lower	7 (7.5)	9 (9.6)
Vocational	33 (35.5)	35 (37.2)
Higher	53 (57.0)	50 (53.2)
No. of objective binge episodes in previous 28 d (SD) [range]	14.6 (10.8) [0-60.0]	16.7 (12.6) [0-62.0]
Global Eating Disorder Examination score, mean (SD)^a^	3.0 (0.9)	2.8 (0.8)
Duration of the eating disorder, mean (SD), y	21.4 (12.8)	23.5 (14.5)
Previous eating disorder treatment		
Yes	11 (12.0)	16 (17.0)
No	81 (88.0)	78 (83.0)
Global Body Shape Questionnaire score, mean (SD)^b^	120.5 (27.9)	126.7 (28.5)
Global Clinical Impairment Assessment score, mean (SD)^c^	21.9 (8.8)	23.1 (9.9)
Eating Disorder Examination-Questionnaire, mean (SD)		
No. of objective binge episodes in previous 28 d	13.8 (9.5)	13.7 (9.5)
Global score^a^	3.4 (1.0)	3.3 (1.0)

Abbreviations: BED, binge-eating disorder; BMI, body mass index (calculated as weight in kilograms divided by height in meters squared); *DSM-5*, *Diagnostic and Statistical Manual of Mental Disorders* (Fifth Edition).

^a^ Scores range from 0 to 6, with higher scores indicating more severe eating disorder pathology.

^b^ Questionnaire used to assess body dissatisfaction. Scores range from 34 to 204, with higher scores indicating more body dissatisfaction.

^c^ Assessment of the severity of personal, social, and cognitive impairment secondary to eating disorder symptoms. Scores range from 0 to 48, with higher scores indicating more severe impairment due to eating disorder symptoms.

Complete measurements at the end of treatment were available for 67 of 93 participants (72.0%) in the web-based guided self-help group and for 77 of 94 (81.9%) in the treatment-as-usual group. At 40 weeks after treatment, complete measurements were available for 67 of 93 participants (72.0%) in the guided self-help group and for 71 of 94 (75.5%) in the treatment-as-usual group. Primary and secondary outcomes, including statistical significance based on 2-sided comparisons of the estimated marginal means between groups, are presented in Table 2.

**Table 2.** Comparative Difference Between Both Groups at Each Assessment

Assessment	Participant Group, No. (95% CI)		Effect size	Contrast estimate (SE) [95% CI]	*P* value
	Web-based–guided self-help (n = 93)	Treatment as usual (n = 94)			
**Objective binge episodes in the previous 28 d**					
End of treatment	3.24 (2.42 to 4.33)	4.06 (3.17 to 5.20)	−0.17	−0.82 (0.53) [−1.86 to 0.21]	.12
20 wk After treatment	1.27 (0.81 to 1.97)	1.84 (1.24 to 2.73)	−0.18	−0.58 (0.42) [−1.41 to 0.26]	.17
**Binge-free days**					
End of treatment	23.84 (22.55 to 25.21)	23.42 (22.34 to 24.55)	0.07	0.42 (0.66) [−0.88 to 1.73]	.52
20 wk After treatment	29.35 (27.06 to 31.85)	28.45 (26.45 to 30.61)	0.08	0.90 (1.45) [−1.95 to 3.75]	.53
**EDE global score** ^a^					
End of treatment	1.43 (1.27 to 1.58)	1.39 (1.24 to 1.54)	0.05	0.04 (0.06) [−0.09 to 0.16]	.55
20 wk After treatment	1.66 (1.48 to 1.85)	1.63 (1.45 to 1.80)	0.04	0.04 (0.11) [−0.18 to 0.26]	.74
**Abstinence from binge eating, %**					
End of treatment	48.27 (36.69 to 60.04)	60.02 (49.07 to 70.06)	NA	−0.12 (0.07) [−0.26 to 0.03]	.11
20 wk After treatment	47.19 (35.71 to 58.97)	44.07 (33.48 to 55.24)	NA	0.03 (0.07) [−0.11 to 0.18]	.67
**Full remission, %**					
End of treatment	39.04 (28.04 to 51.28)	49.89 (38.79 to 61.00)	NA	−0.12 (0.07) [−0.25 to 0.04]	.14
20 wk After treatment	33.45 (23.19 to 45.63)	35.85 (25.88 to 47.22)	NA	−0.02 (0.07) [−0.16 to 0.12]	.74
**EDE-Q, 40 wk after treatment** ^a^					
Objective binge episodes in the past 28 d	2.34 (1.63 to 3.36)	2.44 (1.75 to 3.40)	−0.02	−0.10 (0.54) [−1.16 to 0.96]	.85
Global score	2.17 (1.97 to 2.38)	1.85 (1.66 to 2.05)	0.33	0.32 (0.12) [0.08 to 0.56]	.01
**Body mass index** ^b^					
End of treatment	33.49 (32.82 to 34.16)	34.82 (34.23 to 35.41)	−0.43	−1.33 (0.34) [−2.00 to −0.65]	<.001
20 wk After treatment	33.29 (32.33 to 34.25)	35.10 (34.19 to 36.01)	−0.39	−1.81 (0.60) [−2.99 to −0.62]	.003
**Working Alliance Inventory** ^c^					
End of treatment	154.43 (150.44 to 158.41)	160.42 (156.75 to 164.09)	−0.32	−5.99 (2.33) [−10.57 to −1.42]	.01
**Body Shape Questionnaire** ^d^					
End of treatment	92.88 (88.68 to 97.09)	94.50 (90.65 to 98.34)	−0.08	−1.61 (1.87) [−5.28 to 2.05]	.39
20 wk After treatment	84.18 (79.73 to 88.62)	81.65 (77.54 to 85.76)	0.12	2.53 (2.19) [−1.76 to 6.82]	.25
**Clinical Impairment Assessment** ^e^					
End of treatment	14.12 (12.86 to 15.37)	13.57 (12.43 to 14.72)	0.09	0.54 (0.58) [−0.61 to 1.69]	.35
20 wk After treatment	11.67 (10.36 to 12.98)	9.95 (8.74 to 11.15)	0.28	1.72 (0.63) [0.48 to 2.97]	.01

Abbreviations: EDE, Eating Disorder Examination; EDE-Q, EDE-Questionnaire; NA, not applicable.

^a^ Scores range from 0 to 6, with higher scores indicating more severe eating disorder pathology.

^b^ Calculated as weight in kilograms divided by height in meters squared.

^c^ Inventory that assesses attachment bonds, shared tasks, and shared goals between a participant and therapist. Scores range from 36 to 252, with higher scores indicating stronger therapeutic alliance.

^d^ Questionnaire used to assess body dissatisfaction. Scores range from 34 to 204, with higher scores indicating more body dissatisfaction.

^e^ Assessment of the severity of personal, social, and cognitive impairment secondary to eating disorder symptoms. Scores range from 0 to 48, with higher scores indicating more severe impairment due to eating disorder symptoms.

### Binge-Eating Episodes

A contrast estimate noninferiority was found for the comparative difference in the number of binge-eating episodes in the previous 28 days at the end of treatment (−0.82 [95% CI, −1.86 to 0.21]; *P* = .12) and at 20 weeks after treatment (−0.58 [95% CI, −1.41 to 0.26]; *P* = .17). At the end of treatment, the mean number of binge-eating episodes was in favor of the web-based guided self-help group compared with the treatment-as-usual group (3.24 [95% CI, 2.42-4.33] vs 4.06 [95% CI, 3.17-5.20]). At 20 weeks after treatment, the mean number of binge-eating episodes in the previous 28 days in the web-based guided self-help group was 1.27 [95% CI, 0.81-1.97] compared with 1.84 [95% CI, 1.24-2.73] in the treatment-as-usual group.

### Secondary Outcomes

At the end of treatment and at 20 weeks after treatment, no differences were found in the number of binge-free days. At 40 weeks after treatment, no differences were found in self-reported objective binge-eating episodes in the previous 28 days. No differences were found between groups in eating disorder pathology at the end of treatment or at 20 weeks after treatment; however a significant difference in the contrast estimate was found in favor of treatment as usual on self-reported eating disorder pathology at 40 weeks after treatment (0.32 [95% CI, 0.08-0.56]; *P* = .01). At the end of treatment and at a 20-week follow-up, no differences were found on full remission.

There was no difference in reasons for missing data between groups. No differences in dropout were found; the dropout rate was 22.6% (21 of 93) for web-based guided self-help and 19.1% (18 of 94) for treatment as usual. Sensitivity analyses revealed that participants who dropped out had less educational level (18.0% vs 6.0%), had received previous eating disorder treatment (28.0% vs 11.0%), had more frequent binge eating in the previous 28 days (19.1 [95% CI, 15.1-12.0] vs 14.7 [95% CI, 12.8-16.6]), and had fewer binge-free days (12.3 [95% CI, 9.8-14.8] vs 15.5 [95% CI, 14.13-16.8]).

A difference in the contrast estimate was found in favor of treatment as usual in therapeutic alliance at the end of treatment (−5.99 [95% CI, −10.57 to −1.42]; *P* = .01) and in clinical impairment at 20 weeks after treatment (1.72 [95% CI, 0.48-2.97]; *P* = .01). No differences were found in body-shape dissatisfaction at either time point. Lastly, a difference in the contrast estimate was found in BMI at the end of treatment (−1.33 [95% CI, −2.00 to −0.65]; *P* < .001) and at the 20-week follow-up (−1.81 [95% CI, −2.99 to −0.62]; *P* = .003), and those in the treatment-as-usual group increased in BMI, while those in the web-based guided self-help group kept a stable BMI. In the web-based guided self-help group, 1 adverse event occurred, while in the treatment-as-usual group, 3 adverse events occurred. Details on dropout, care consumption, therapist adherence, and adverse events are provided in the eResults in Supplement 2.

### Sensitivity Analysis

As a sensitivity analysis, a per-protocol analysis (ie, participants who dropped out of treatment excluded) was performed. A contrast estimate noninferiority was found on the number of objective binge-eating episodes in the previous 28 days at the end of treatment (−0.92 [95% CI, −1.98 to 0.14]; *P* = .09) and at 20 weeks after treatment (−0.61 [95% CI, −1.50 to 0.28]; *P* = .18). The outcomes were comparable with the intention-to-treat analysis, with the exception that noninferiority was not found on the number of binge-eating episodes in the previous 28 days at 40 weeks after treatment, and no significant difference was found in the therapeutic alliance at the end of treatment. Complete findings from the sensitivity analysis are provided in eTable 2 in Supplement 2.

## Discussion

This randomized clinical trial aimed to examine web-based guided self-help compared with treatment as usual for adults with BED. The findings demonstrate that web-based guided self-help is noninferior to treatment as usual in reducing the number of binge-eating episodes at the end of treatment and at 20 weeks after treatment. Participants in the guided self-help group had a comparable decrease in binge-eating episodes with those in the treatment-as-usual group despite only having 12 weeks of treatment with less therapist support. Although there was stronger therapeutic alliance in the treatment-as-usual group at the end of treatment, this does not appear to be a barrier to successful treatment effect, suggesting that neither session length nor scripted protocol adherence influenced treatment outcomes.^41^

These findings align with prior work supporting the benefits of guided self-help in eating disorders^40,42^ and other psychiatric disorders.^22,23,43^ In contrast to earlier findings,^13^ there were no significant differences in treatment dropout between groups, which represents an encouraging finding for future developments in web-based mental health care. Of 207 patients assessed for eligibility, only 8 (3.9%) refused to participate in the trial, highlighting patient willingness to be randomized to receive a shorter treatment with less therapist contact, requiring more self-management. Furthermore, there was only 1 adverse event in the web-based guided self-help group, suggesting that the novel treatment is safe. This study contributes to the growing evidence supporting not only guided self-help treatments but also web-based therapies as standalone treatments that have the potential to improve access to care in the Netherlands and more globally. With significantly fewer therapist hours compared with standard treatment, these findings can support the global challenges of staff shortages and long waiting lists in mental health care.^44^

### Strengths and Limitations

The present study has several strengths. First, it adhered to a strict protocol^36,45^ to directly compare web-based guided self-help with comprehensive treatment as usual. Second, it was well-powered, and all measures were conducted by assessors blinded to the allocation group. Third, both treatments were offered through videoconferencing, which enhanced the comparability between groups. Additionally, participants who took part in the study were representative of treatment-seeking adults with BED.

This study also has limitations. One of the study’s limitations is the lack of interview data available at 40 weeks after treatment. Additionally, there was no closed posttreatment period to ensure that participants did not begin any other treatments. Given the exploratory nature of secondary analyses, no formal adjustments for multiple testing were applied; therefore, findings should be interpreted with caution due to the risk of type I error. As treatment was within the context of a specialized eating disorder center with trained specialists, future work should aim to replicate this method in other settings and within a more heterogeneous group. Participants included in this trial were all referred for specialized eating disorder treatment, which may limit the applicability of these findings to other nonclinical populations that may also struggle with binge eating.

## Conclusions

In this randomized clinical trial examining the comparative difference between web-based guided self-help and treatment as usual, the findings suggest that web-based guided self-help can be a viable treatment for adults with BED. The many advantages of this noninferior, scalable, web-based treatment intervention, which requires minimal therapeutic involvement, should be studied further. Based on the positive findings from this study, future work should examine the source of guidance in the guided self-help program. Understanding the comparative differences in who administers guidance, including nonspecialized health care workers and experts by experience, will provide more evidence for the accessibility and scalability of treatment.

## Notes

Supplement 1.
Trial Protocol

Supplement 2. **eTable 1.** Secondary Measures **eMethods.** Details of the Main Statistical Analyses
eResults.
**eTable 2.** Per-Protocol Analysis of Comparative Differences Between Groups per Assessment

Supplement 3.
Data Sharing Statement
