# Supplementary material for: Web-Based Guided Self-Help vs Treatment as Usual for Binge-Eating Disorder: A Randomized Clinical Trial
Source: JAMA Netw Open. 2025 Oct 10;8(10):e2536644. doi: 10.1001/jamanetworkopen.2025.36644 (PMC12514623; doi:10.1001/jamanetworkopen.2025.36644)
Supplement: Supplement 1. — Trial Protocol [file jamanetwopen-e2536644-s001.pdf]

RESEARCH PROTOCOL

Online Cognitive behavioral therapy-Enhanced: guided self-help versus screen to screen for Binge eating disorder, A Randomized Controlled Trial

PROTOCOL TITLE Online Cognitive Behavioral Therapy – Enhanced: guided self-help versus screen-to-screen for Binge Eating Disorder, A Randomized Controlled Trial

**Protocol ID**	NL 76368.100.21
**Short title**	Online begeleide zelfhulp/ volledige behandeling voor eetbuistoornis
**EudraCT number**	Not applicable
**Version**	1.0
**Date**	28-2-2021
**Coordinating investigator/project leader**	Prof. Dr. J. Dekker<br>Head of Research<br>Arkin dept. Research<br>Klaprozenweg 111, 1033 NN Amsterdam<br>PO Box 75848, 1070 AV Amsterdam<br>*E* jack.dekker@arkin.nl<br>*T* +31 20 590 5102
**Principal investigator(s) (in Dutch: hoofdonderzoeker/ uitvoerder)**	Elske van den Berg<br>Clinical director, Clinical psychologist<br>Novarum, centrum voor eetstoornissen<br>Laan van de Helende meesters 2, 1186 AM Amstelveen<br>E: elske.vanden.berg@novarum.nl<br>T +31 20 590 4714<br><br>Bernou Melisse<br>Novarum, centrum voor eetstoornissen<br>Laan van de Helende meesters 2, 1186 AM Amstelveen<br>E: bernou.melisse@novarum.nl<br>T: +31 6- 21958289
**Sponsor (in Dutch: verrichter/opdrachtgever)**	Novarum, centrum voor eetstoornissen Amsterdam<br>Laan van de Helende Meesters 2, 1186 AM Amstelveen
**Subsidizing party**	Arkin Mental Health Care institute<br>Klaprozenweg 111, 1033 NN Amsterdam<br>PO Box 75848, 1070 AV Amsterdam
**Independent expert (s)**	Laura van Riel, MD/ psychiatrist<br>Domselaerstraat 128 ,1093 MB Amsterdam<br>020 - 590 5201

**Laboratory sites**	Not applicable
**Pharmacy**	Not applicable

PROTOCOL SIGNATURE SHEET

Name	Signature	Date
**Head of Department:**<br>Prof. Dr. J. Dekker<br>Head of Research<br>Arkin dept. Research<br>Klaprozenweg 111, 1033 NN Amsterdam<br>PO Box 75848, 1070 AV Amsterdam<br>E jack.dekker@arkin.nl<br>T +31 20 590 5102	[signature]	24/03/21
**Coordinating Investigator/Project leader/Principal Investigator:**<br>Elske van den Berg<br>Clinical director, Clinical psychologist<br>Novarum, centrum voor eetstoornissen<br>Laan van de helende meesters 2, 1186 AM Amstelveen<br>E: elske.vanden.berg@novarum.nl<br>T +31 20 590 4714	[signature]	1/3/21
**Coordinating Investigator/Project leader/Principal Investigator:**<br>Bernou Melisse<br>Novarum, centrum voor eetstoornissen<br>Laan van de helende meesters 2, 1186 AM Amstelveen<br>E: bernou.melisse@novarum.nl<br>T: +31 6- 21958289	Bernou	1-3-'21

TABLE OF CONTENTS

1.INTRODUCTION AND RATIONALE 9
2.2 OBJECTIVES 11
3.STUDY DESIGN 12
4.STUDY POPULATION 15
4.1Population (base) 15
4.2Inclusion criteria 15
4.3Exclusion criteria 15
4.4Sample size calculation 15
5.TREATMENT OF SUBJECTS 16
5.1Experimental treatment, Guided self-help CBT-E 16
5.1Treatment as usual, Screen-to-screen CBT-E 17
5.1Use of co-intervention (if applicable) 18
5.1Escape medication (if applicable) 18
6.INVESTIGATIONAL PRODUCT 18
7.NON-INVESTIGATIONAL PRODUCT 18
8.METHODS 18
8.1Study parameters/endpoints 18
8.1.1Main study parameter/endpoint 18
8.1.2Secondary study parameters/endpoints 19
8.1.3Secondary study parameters/endpoints (if applicable) 20
8.1.4Other study parameters: 20
8.2Randomization, blinding and treatment allocation 21
8.3Study procedures 21
8.4Withdrawal of individual subjects 22
8.4.1Specific criteria for withdrawal (if applicable) 22
8.5Replacement of individual subjects after withdrawal 22
8.6Follow-up of subjects withdrawn from treatment 23
8.7Premature termination of the study 23
9.SAFETY REPORTING 23
9.1Temporary halt for reasons of subject safety 23
9.2AEs, SAEs and SUSARs 23
9.2.1Adverse events (AEs) 23
9.2.2Serious adverse events (SAEs) 23
9.2.3Suspected unexpected serious adverse reactions (SUSARs) 24
9.3Annual safety report 24
9.4Follow-up of adverse events 24
9.5[Data Safety Monitoring Board (DSMB) / Safety Committee] 24
10.STATISTICAL ANALYSIS 24
10.1Primary study parameter(s) 24
10.2Secondary study parameter(s) 25
10.3Secondary study parameter(s) 25

10.4Study parameters 26
10.5Interim analysis (if applicable) 28
11.ETHICAL CONSIDERATIONS 28
11.1Regulation statement 28
11.2Recruitment and consent 28
11.3Objection by minors or incapacitated subjects (if applicable) 29
11.4Benefits and risks assessment, group relatedness 29
11.5Compensation for injury 29
11.6Incentives 30
12.ADMINISTRATIVE ASPECTS, MONITORING AND PUBLICATION 30
12.1Handling and storage of data and documents 30
12.2Monitoring and Quality Assurance 30
12.3Amendments 30
12.4Annual progress report 31
12.5Temporary halt and (prematurely) end of study report 31
13.STRUCTURED RISK ANALYSIS 32
14.REFERENCES 33
14.1.1Welkom! 35
14.2Wat ga ik doen? 35
14.3Mijn planning 36
14.4Hoe vul ik de eetregistratie in? 36
8.4.1Alternatieven voor eetbuien 37
8.4.2Je voorbereiden op het inzetten van alternatieve activiteiten 37
14.5Mijn lijst van activiteiten 37
8.5.1Probleemoplossing 38
14.6Oefenen 38
8.6.1Eetregelmodule 38
14.7Stap 1. Achterhaal welke voedingsmiddelen je vermijdt 38
14.8Stap 2. Verdeel de voedingsmiddelen in categorieën 39
14.9Stap 3. Introduceer de voedingsmiddelen in je dieet 39
14.10En onthoud 39
8.10.1Lichaamsbeeldmodule 40
14.11Aan de slag 40
8.11.1Gebruik van de spiegel 40

LIST OF ABBREVIATIONS AND RELEVANT DEFINITIONS

AE	Adverse Event
AR	Adverse Reaction
BED	Binge Eating Disorder
BMI	Body Mass Index
CA	Competent Authority
BSQ	Body Shape Questionnaire
CBT-E	Cognitive Beahavioral Therapy - Enhanced
CIA	Clinical Impairment Assessment
CCMO	Central Committee on Research Involving Human Subjects; in Dutch: Centrale Commissie Mensgebonden Onderzoek
DSMB	Data Safety Monitoring Board
ED	Eating Disorder
EDE	Eating Disorder Examination
EDE-Q	Eating Disorder Examination- Questionnaire
EU	European Union
EudraCT	European drug regulatory affairs Clinical Trials
GP	General practicioner
IC	Informed Consent
IMP	Investigational Medicinal Product
IMPD	Investigational Medicinal Product Dossier
METC	Medical research ethics committee (MREC); in Dutch: medisch-ethische toetsingscommissie (METC)
OFSED	Other Specified Feeding or Eating Disorder
RCT	Randomized Controlled Trial
(S)AE	(Serious) Adverse Event
SH	Self- Help
SPC	Summary of Product Characteristics; in Dutch: officiële productinformatie IB1-tekst
Sponsor	The sponsor is the party that commissions the organisation or performance of the research, for example a pharmaceutical company, academic hospital, scientific organisation or investigator. A party that provides funding for a study but does not commission it is not regarded as the sponsor, but referred to as a subsidising party.
SUSAR	Suspected Unexpected Serious Adverse Reaction
TAU	Treatment as usual
TiC-P	Costs associated with Psychiatric illness
UAVG	Dutch Act on Implementation of the General Data Protection Regulation; in Dutch: Uitvoeringswet AVG
WAI	Working Alliance Inventory
WMO	Medical Research Involving Human Subjects Act; in Dutch: Wet Medisch-wetenschappelijk Onderzoek met Mensen

SUMMARY

Rationale: Cognitive Behavioral Therapy- Enhanced (CBT-E) is an evidence-based treatment for all eating disorders. Offering treatment remotely has several advantages for the patient such as removal of geographical barriers, sessions can be held within the patients safe environment, they can communicate with their therapist wherever they are, and reduced travel costs and travel time. The Covid-19 pandemic increased the urgence of offering specialized treatment remotely and several outpatient centers introduced potential adaptations to their existing levels of care, including CBT-E. There is a lack of studies examining remote versions of CBT-E and no study has directly compared efficacy of guided self-help CBT-E with screen-to-screen CBT-E and investigated the effect of treatment dose. At Novarum center for eating disorders in the Netherlands both CBT-E treatments will be offered online. One treatment protocol will be developed involving a screen-to-screen version of CBT-E (screen-to-screen CBT-E) and another one involves a guided self-help treatment protocol (guided self-help CBT-E). It's hypothesized that screen-to-screen CBT-E is superior to guided self-help CBT-E and, that guided self-help CBT-E is superior in terms of cost-efficacy.

Objective: This study compares the effectiveness of guided self-help CBT-E with to screen-to-screen CBT-E in terms of robust remission at end of treatment and during follow up. Robust remission will be defined as eating disorder pathology below a clinical cut-off and no binge eating pathology. Secondary objective is to measure the effectiveness with regard to clinical impairment and quality of life of guided self-help CBT-E in comparison with screen-to-screen CBT-E group after treatment and during follow-up and, to investigate the moderating effect of severity of body dissatisfaction and the covariating effect of early menarche, as early menarche is expected to be associated with body dissatisfaction.

Study design: A single center randomized controlled trial assessing the effects of the newly developed screen-to-screen CBT-E compared with guided self-help CBT-E. Both treatments are based on Cognitive Behavioral Therapy- Enhanced treatment protocol and will be offered online. Stratification will take place based on BMI group; $19.5 \leq BMI \leq 35$ or $35 < BMI \leq 40$. 180 participants (142= without correction) will participate in this study. Parameters will be measured at start of treatment (week 0), week 5, end of treatment (week 12 for guided self-help, week 20 for screen-to-screen CBT-E), follow up measurements are 20and 60 weeks after end of treatment.

Study population: Study population exists of 180 participants diagnosed with Binge Eating Disorder (BED) and Other Specified Feeding or Eating Disorder (OSFED) BED (OSFED BED. All participants were referred to Novarum Center for Eating Disorders by their General Practitioner or other mental health care institutions in order to seek treatment for their eating disorder. Participants are aged ≥ 18 years.

Intervention: Cognitive Behavorial Therapy- Enhanced (CBT-E) is an evidenced based treatment for eating disorders. Treatment period is 20 weeks, including 20 sessions of 50 minutes each. The first 4 weeks will involve 8 sessions, weeks 5-14 involve weekly sessions and week 15-20 involves bi-weekly sessions. Sessions will be conducted in a screen-to-screen setting. Guided self-help CBT-E is an online guided self-help version of CBT-E based on the self-help book "Overcoming Binge Eating". Treatment period is 12 weeks and patients will complete exercises at an online treatment platform on a daily basis. Once a week they will have a therapy session of 20 minutes offered through video call.

Main study parameters/endpoints: The main study parameter is to compare the relative treatment efficacy of guided self-help CBT-E versus screen-to-screen CBT-E, reported as

robust remission pre- and post-treatment and during follow-up. The primary parameter will be measured through the Eating Disorder Examination (EDE) and Eating Disorder Examination Questionnaire (EDE-Q) at start and end of treatment and 60 weeks follow up, during follow up, 20, weeks post treatment (week 40) by the EDE-Q. Secondary parameters are the efficacy with regard to clinical impairment and quality of life of guided self-help CBT-E in comparison with screen-to-screen CBT-E after treatment and during follow-up and, to investigate the moderating effect of severity of body dissatisfaction and the association of early menarche and body dissatisfaction. Secondary parameters involving quality of life, clinical impairment and body dissatisfaction will be measured at start and end of treatment, during follow up (20, 60 weeks post treatment) by the EQ-5D-NL, Clinical Impairment Assessment (CIA) and, Body Shape Questionnaire (BSQ) . The association between body dissatisfaction and early menarche will be measured at start of treatment. Other parameters are the moderating effects of therapeutic alliance between both conditions and the compare cost- efficacy. Therapeutic alliance will be measured by the Working Alliance Inventory and costs by the questionnaire on Costs associated with Psychiatric illness (TiC-P) during week 5, 12 and at end of treatment.

Nature and extent of the burden and risks associated with participation, benefit and group relatedness: Participants burden is expected to be limited since they receive either screen-to-screen CBT-E or guided self-help CBT-E which is less extensive. Participants will be requested to complete questionnaires at baseline, during and at end of treatment which will be used as treatment evaluation and are therefore also included without study participation. Completion of the self-report follow up measures will be 30 minutes each, 60 minutes in total to complete all follow-up measures. Completion of the interviews will take 3 times 45-60 minutes. In total a burden of maximum 180 minutes for each participant in comparison with treatment without study participation. There are minimal risks associated with participation in this study as the medical device is classified as a class 1 medical device.

1. INTRODUCTION AND RATIONALE

Eating disorders have a significant impact on the psychological, social and physical well-being of affected patients (Hay, Girosi, & Mond, 2015; Rojo-Moreno et al., 2015) including anxiety, depression (Watson, Allen, Fursland, Byrne, & Nathan, 2012), and high rates of mortality (Smink, van Hoeken, & Hoek, 2012). Eating disorders are characterized by over-evaluation of shape and weight (Fairburn & Harrison, 2003) and have a life-time prevalence in the Netherlands of 1.74% (Preti et al., 2009). On the basis of extensive research binge eating disorder (BED) was acknowledged as a distinctive eating disorder by the DSM 5 (Association, 2013). BED is specifically characterized by recurrent episodes of binge eating accompanied by a sense of lack of control and absence of compensatory behavior. The binges occur at least once a week and, if they are less frequent, the individual is classified with other specified feeding or eating disorder (OSFED) BED (Association, 2013; Mustelin, Lehtokari, & Keski-Rahkonen, 2016). BED is the most common eating disorder with an estimated life-time prevalence of 2% (Kessler et al., 2013), however, the prevalence is estimated to be higher among the obese population (Kessler et al., 2013; Van der Horst, Van Os, van der Horst, & van Elburg, 2019).

There are several kind of treatments for BED available, however Cognitive Behavioral Therapy (CBT) is the most well established treatment with high recovery rates (Hilbert et al., 2019). "Cognitive Behavioral Therapy- Enhanced" (CBT-E) is specifically developed for eating disorders and an evidence based treatment for all eating disorders (Australian & Psychiatrists, 2014; Fairburn et al., 2015; Health & Excellence, 2017). According to efficacy trials conducted by professor Fairburn, developer of CBT-E (Fairburn et al., 2015; Fairburn et al., 2009), recovery rates of CBT-E are between 50- 68% in transdiagnostic samples, and an effectiveness study showed a recovery rate 65.2% for BED (Melisse, Jonge, et al., 2021).

There is a constant need to increase patient's access to specialized eating disorder treatment (Abrahamsson, Ahlund, Ahrin, & Alfonsson, 2018). Offering treatment remotely has several advantages for the patient such as removal of geographical barriers, sessions can be held within the patients safe environment, they can communicate with their therapist wherever they are, and reduced travel costs and travel time (Abrahamsson et al., 2018; Becker, Hadley Arrindell, Perloe, Fay, & Striegel-Moore, 2010; Evans et al., 2011; van den Berg et al., 2020). In addition, the Covid-19 pandemic increased the urgence of offering specialized treatment remotely (Termorshuizen et al., 2020) and several outpatient centers introduced potential adaptations to their existing levels of care, including CBT-E, (Murphy, Calugi, Cooper, & Dalle Grave, 2020; Waller et al., 2020). Examples of such adaptations are offering CBT-E online, either as guided self-help (van den Berg et al., 2020) or as a screen-to-screen treatment through video call (Abrahamsson et al., 2018; Murphy et al., 2020; Waller et al., 2020).

During screen-to-screen treatment a secure video-link is established between patient and therapist (Abrahamsson et al., 2018). Screen-to-screen therapy has several advantages, for example, non-verbal communication aspects, such as gestures, body posture and facial expression, are barely impacted (Myers & Turvey, 2013). Another advantage is that patient and therapist have the possibility to exchange documents (Abrahamsson et al., 2018), while it does not compromise treatment dose offered to the patient (Murphy et al., 2020). Screen-to-screen therapy is effective (Arnberg et al., 2014) and treatment outcomes are not compromised in comparison with face-to-face treatment (Mitchell et al., 2008). However, only a single case multiple baseline study examined treatment outcomes of screen-to-screen CBT-E, but eating disorder pathology decreased significantly (Abrahamsson et al., 2018).

Screen-to-screen treatment appears to be a suitable alternative for face-to-face treatment however, international guidelines recommend to offer guided self-help for patients suffering from BED following the stepped care principle (Australian & Psychiatrists, 2014; Health & Excellence, 2017). Offering guided self-help has several advantages (van den Berg et al., 2020). There is a lack of specialized therapists for eating disorder treatment in general, leading to patients having to wait longer than preferred for specialized treatment (Shafran et al., 2009). Guided self- help may reduce the amount of intensive therapy and will therefore reduce waitinglist duration due to less time of a specialist's invested in a single treatment (Fairburn & Peveler, 1990; Shafran et al., 2009). Online guided self-help treatments include usage of apps on different devices such as smartphones or computer (Abdullah et al., 2016) in combination with therapeutic support. However, studies reporting efficacy of guided self-help CBT-E are scarce. Only one study reported that guided self-help version of CBT-E showed greater reduction in number of binge eating episodes and overall eating disorder pathology than the waitinglist condition (Melisse, Berg, et al., 2021).

There is a lack of studies examining remote versions of CBT-E and no study has directly compared efficacy of guided self-help CBT-E with screen-to-screen CBT-E and investigated the effect of treatment dose. In addition, several studies who compared face-to-face CBT with guided self-help CBT found equally efficacy in terms of reduction in binge eating and eating disorder pathology (Mitchell et al., 2008; Wilson, 2011) while other studies found greater efficacy of CBT (de Zwaan et al., 2017; Zerwas et al., 2017) but efficacy was comparable at 12 months follow-up (Zerwas et al., 2017). Another study found comparable efficacy in reduction of binge eating behavior but guided self-help was inferior in terms of reduction in over-evaluation of shape and weight (Paxton, McLean, Gollings, Faulkner, & Wertheim, 2007).

As the role of treatment dose when offering eating disorder treatment is unclear, determining the moderating effects of screen-to-screen CBT-E compared to guided self-help CBT-E may enhance the development of personalized medicine (Kraemer, 2013). Although studies investigating moderation of eating disorder treatment are scarce, it is assumed that the severity of body dissatisfaction moderates treatment outcome (Grilo, 2017; Grilo, Masheb, & Crosby, 2012; Stice, 2002). Grilo (2012) (Grilo et al., 2012) concluded that "overevaluation was the most salient moderator of treatment outcome. In addition, in 2017 Grilo, stated that BED patients with higher levels of overevaluation of shape and weight, and thereore greater body dissatisfaction improve less from treatment than BED patients with lower levels or no overvaluation at all. Differences in improvement were mainly significant in reduction of binge eating episodes (Grilo, 2017). It has been suggested that early pubertal development may foster body image and eating disturbances; as early menarche leads to increased adipose tissue, it moves girls away from the normative body shape of adolescents which theoretically increases body dissatisfaction and consequent dieting and eating disturbances. Early menarche might therefore serve as a covariate. Findings however are inconclusive for the claim that early menarche is a risk factor for body dissatisfaction and eating pathology (Mitchison & Hay, 2014). These inconclusiveness may be related to the fact that the adverse effects of early menarche are developmentally localized and may dissipate over time (Stice, 2002). For exploratory purposes the presence of early menarche will be assessed, in order to examine a possible association with body dissatisfaction.

At Novarum center for eating disorders in the Netherlands two versions of CBT-E will be offered, both online. One treatment protocol will be developed involving a screen-to-screen version of CBT-E (screen-to-screen CBT-E) and another one involves a guided self-help treatment protocol (guided self-help CBT-E) based on the book "Overcoming binge eating"

(Fairburn, 2013; Fairburn, 2016). The primary aim of this study is to examine the efficacy of guided self-help CBT-E in comparison with screen-to-screen CBT-E. Efficacy will be studied by monitoring eating disorder symptoms pre and post treatment. Clinical outcomes are reduction in binge eating episodes (Lynch et al., 2010) and eating disorder pathology (Cooper, Cooper, & Fairburn, 1989; Fairburn & Beglin, 1994; Fairburn & Harrison, 2003) defined as robust remission. Secondary aim is to examine the efficacy with regard to clinical impairment and quality of life between guided self-help CBT-E in comparison with screen-to-screen CBT-E group after treatment and during follow-up and, to investigate the moderating effect of severity of body dissatisfaction. Early menarche and body dissatisfaction are expected to be associated and therefore early menarche is expected to be o covariate in treatment outcome. Tertiary aim is to calculate cost efficacy between both groups. Efficacy will be examined through a parallel group randomized controlled trial.

It's hypothesized that both groups display significant decreases in number of binge eating episodes and eating disorder pathology and therefore report robust remission (Berg et al., 2020; Bulik, Marcus, Zerwas, Levine, & La Via, 2012; Melisse, Jonge, et al., 2021). However, expected is that guided self-help CBT-E is inferior to screen-to-screen CBT-E post treatment but non- inferior during follow-up (Bulik et al., 2012). In addition, cost efficacy of guided self-help CBT-E is expected to be superior to screen-to-screen CBT-E. Furthermore, greater body dissatisfaction is expected to result in poorer treatment outcome, while greater body dissatisfaction is expected to be associated with early menarche. Furthermore, guided self-help CBT-E is expected to be more cost- effective than screen-to-screen CBT-E (Bouwmans et al., 2013). In addition, in case guided self-help CBT-E is found to be less effective than screen-to-screen CBT-E it might be an acceptable treatment solution in terms of cost-effectiveness for patients with less severe eating disorders.

2. 2 OBJECTIVES

2.1 Primary Objective:

The primary objective of the RCT is to examine the efficacy of guided self-help CBT-E compared to screen-to-screen CBT-E. Efficacy will be assessed by the Eating Disorder Examination (EDE) (Cooper & Fairburn, 1987). Reduction in number of objective binges and eating disorder pathology will be assessed by the Eating Disorder Examination (EDE) (Cooper & Fairburn, 1987). Eating disorder pathology is defined as restraint eating, dieting, shape concern and weight concern. Follow-up measures will be assessed by the Eating Disorder Examination Questionnaire (EDE-Q) at week 40 (20 weeks follow-up) (Fairburn & Beglin, 2008) and by the EDE (Cooper & Fairburn, 1987) in week 80 (60 weeks follow-up).

2.2 Secondary and tertiary Objective(s):

Secondary objective is to measure the efficacy with regard to clinical impairment and quality of life of guided self-help CBT-E in comparison with screen-to-screen CBT-E group after treatment and during follow-up (20 and, 60 weeks post-treatment) and, to investigate the moderating effect of severity of body dissatisfaction and the covariating effect of early menarche. Clinical impairment will be assessed by the Clinical Impairment Assessment (Bohn et al., 2008) and quality of life by the EQ-5D-5NL (Versteegh et al., 2016). The severity of body dissatisfaction will be measured by the body shape questionnaire (BSQ) (Cooper, Taylor, Cooper, & Fairbum, 1987). Participants will be asked their age of onset of

menarche and will be asked whether they experienced developmental deviance at time of onset.

In addition, an economic evaluation will be performed alongside the RCT in line with ISPOR 2015 guidelines (Ramsey et al., 2015), to test for cost- efficacy of guided self-help CBT-E in comparison with screen-to-screen CBT-E. Costs and effects (Bouwmans et al., 2013) will be measured at start, end of treatment and at 20and, 60 weeks post treatment. See table 1 for a measurement time table.

Table 1. Overview of planned assessments

	Timing during CBT-E	
Treatment phase	Guided self-help	Screen-to-screen
T0: baseline assessment	week 0	week 0
T1 : treatment evaluation	week 5	week 5
T2: end of treatment	week 12	week 20
T3: 20 weeks post treatment	week 32	week 40
T4: 60 weeks post treatment	week 72	week 80

3. STUDY DESIGN

A single center randomized controlled trial assessing the effects of screen-to-screen CBT-E compared with guided self-help CBT-E. Both treatments are based on Cognitive Behavioral Therapy- Enhanced treatment protocol and will be offered online. Stratification will take place based on BMI group; 19.5 ≤ BMI ≤ 35 or 35< BMI ≤ 40.

Assessments will take place at baseline (before randomization) and 4times during and after treatment. Assessments will synchronous in treatment phase but will not be equal between the two conditions in terms of time. In case of guided self-help CBT-E this will be at start of treatment, at week 5 which is the evaluation-point of treatment, end of treatment (week 12), 20 weeks after treatment (week 32and 72 weeks after treatment (week 80). In case of CBT-E this will be at start of treatment, at week 5 which is the evaluation-point of treatment, , end of treatment (week 20), 20 weeks after treatment (week 40), and, 60 weeks after treatment (week 80). See Table 1 and Figure 1. In addition, the placement of time of these measurements will be synchronized. The trial will be registered in the Netherlands Trial Register after METc approval. Study duration, including follow up measures will be 4 years (48 months). Participants will be recruited at Novarum (Amstelveen, the Netherlands), the eating disorder department of Arkin.

Figure 1. Flowchart

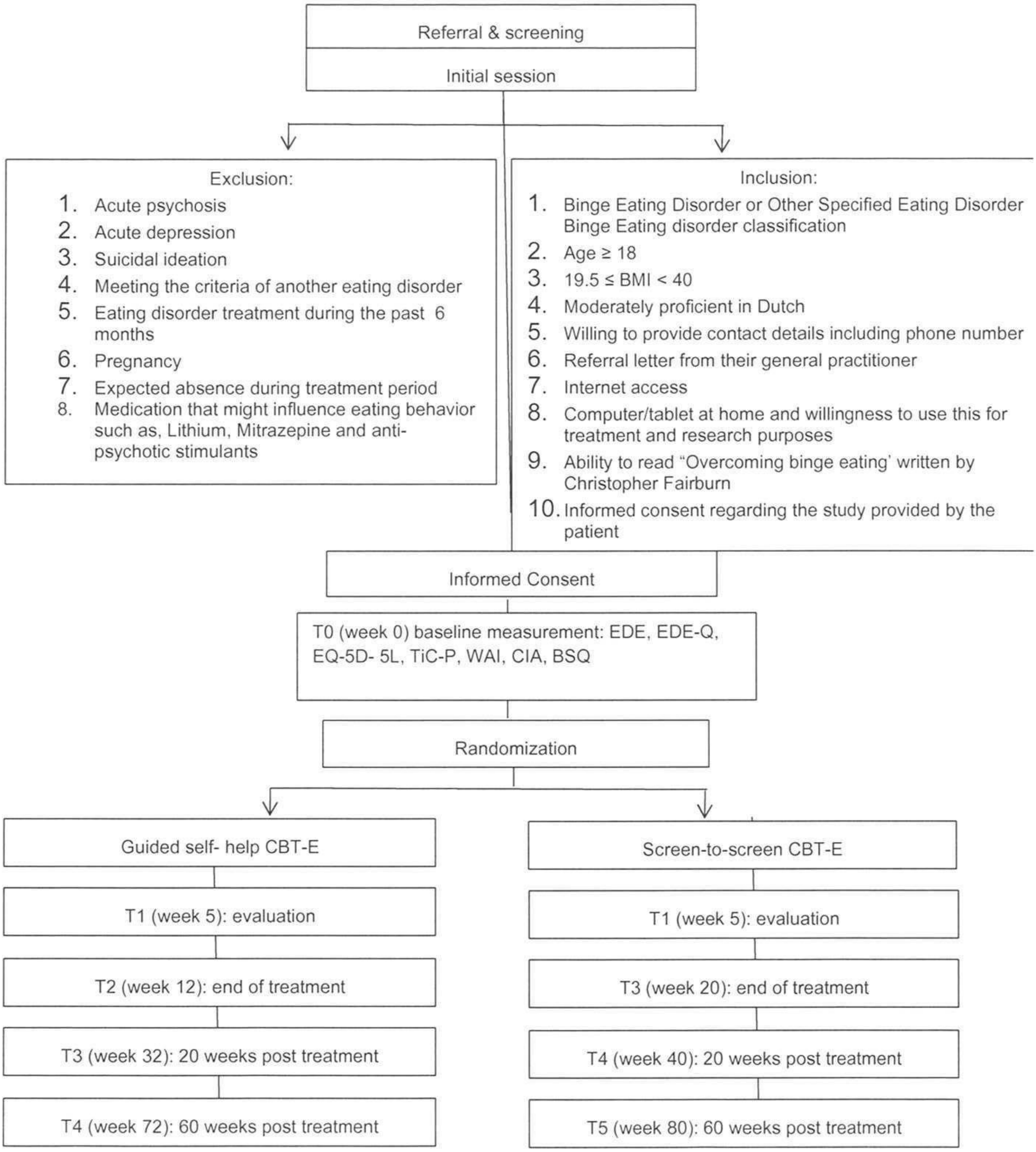

4. STUDY POPULATION

Population (base)

The research population consists of patients, aged ≥18, with a diagnosis of binge eating disorder (BED) or other specified feeding or eating disorder (OSFED) binge eating disorder (Association, 2013). Patients are enrolled at Arkin/ Novarum, Center for Eating Disorders in Amstelveen, the Netherlands.

Inclusion criteria

In order to be eligible to participate in this study, a participant must meet all of the following criteria:

1. binge eating disorder or other specified feeding or eating disorder (OSFED) binge eating disorder classification
2. Age ≥ 18
3. 19.5 ≤ BMI < 40
4. Moderately proficient in Dutch
5. Willing to provide contact details including (mobile)phone number
6. Referral letter from their general practitioner (GP)
7. Internet access
8. Computer/tablet at home and willingness to use this for treatment and research purposes
9. Ability to read "Overcoming binge eating' written by Christopher Fairburn
10. Informed consent regarding the study provided by the patient

Exclusion criteria

A potential subject who meets any of the following criteria will be excluded from participation in this study:

1. Acute psychosis
2. Acute depression
3. Suicidal ideation
4. Anorexia Nervosa or Bulimia Nervosa
5. Treatment for an eating disorder during the past 6 months
6. Pregnancy
7. Expected absence during treatment period
8. Medication that might influence eating behavior such as, Lithium, Mitrazepine and anti-psychotic stimulants

Sample size calculation

CBT-E and guided self-help CBT-E are effective treatments for binge eating disorder. Treatment dose is defined as total time of therapy offered (Delgadillo 2019). As screen-to-screen CBT-E involves a greater treatment dose than guided self-help CBT-E. Treatment dose in guided self-help CBT-E will be 144 minutes and 1000 minutes in screen-to-screen CBT-E. Therefore a difference in efficacy will be expected between both conditions. In order to detect an effect size of d=0.47, N= 144 (n= 72 per arm) patients need to be included. Self-

help treatment for BED has an overall drop-out of 24% (Hilbert et al., 2019) and efficacy studies of CBT-E reported drop out rates of 20% (Fairburn et al., 2015; Fairburn et al., 2009). Therefore 25% more participants should be included in order to correct for drop-out. The sample size without correction is *N*= 144 (*n*= 72 per arm). The sample size corrected for drop- out will be *N*= 180 (*n*=90 per arm). Sample size calculation is done using R package 'pwr'.

5. TREATMENT OF SUBJECTS

All participants will receive online CBT-E. However, the experimental group will receive guided self-help CBT-E and the control group will receive screen-to-screen CBT-E.

5.1 Experimental treatment, Guided self-help CBT-E

Guided self-help CBT-E is an online version of CBT-E (Fairburn, 2008; Fairburn, Cooper, & Shafran, 2003) Christopher G Fairburn, 1995; C.G.) and is developed by specialists at Novarum Center for Eating Disorders. A software team implemented this into a website and application. During the pilot phase, development on the app was an interactive process involving patients and therapists feedback on user friendliness, ease of navigation lay-out etc. After patients get referred by their GP they have an intitial intake session in order to be assessed. During their advisory session they will be informed about guided self-help CBT-E treatment. Before patients are eligible to start treatment they have to read part one of the book 'Overcoming binge eating' (Christopher G Fairburn, 1995). guided self-help CBT-E is a 12 week program. Patients have to start to read information online, monitor their eating behavior, schedule weighing once a week and schedule 2 self-evaluation sessions per week. A few days after start of treatment they have a 20 minutes video call session with their therapist. These sessions are pre-scripted in order to ensure consistency between therapists
During the first 4 weeks patients have to monitor their eating behavior, including their thoughts and feelings and establish a regular eating pattern. They also have to introduce alternative activities for binge eating and will work on their problem solving skills. During week five they complete assessment questionnaires and the therapist and patient evaluate the patients progression. Based on this session they decide to add a module on shape concern or dietary restraint during week 6-11 on top of monitoring, regular eating, problem solving and alternatives for binge eating. Before the 12th session patients have to complete the questionnaires. Results are discussed during session 12 and they discuss what to do in order to prevent set-backs.

Step	Focus	Period (12 weeks in total)
Phase 1, Step 1	Starting well	1 week
Step 2	Establish a regular eating pattern	1 week

Step 3	Alternatives for binge eating	1 week
Step 4	Problem solving	1 week
Phase 2, Step 5	Evaluation	1 week
Phase 3, Step 6	Module: dietary restraint	Distributed over 6 weeks
Step 6	Module: shape concern	Distributed over 6 weeks
Phase 4, Step 8	Ending well	1 week

Therapists are able to activate an account and patients are able to log in in their digital environment through my.karify.com. They have to register with a personal username and password. Patients as well as their therapists are able to access the intervention anytime. Once the patient completes an assignment the therapist receives a notification by email and has the possibility to access the assignments. All therapists are skilled CBT-E therapists, who completed the CBT-E training developed by Centre for Research on Eating Disorders at Oxford (CREDO) (Fairburn, 2008). Therapists have different disciplinary backgrounds and completed a post doc degree (clinical psychologist), masters degree (psychologist) or a bachelors degree (dieticians and social workers). A manual is available for all guided self-help CBT-E therapists. All intervention modules are explained in detail. CBT-E treatment will not be altered or interfered with during the study. Guided self-help CBT-E is a 100% guided self- help treatment without face to face sessions and will not be blended with screen-to-screen CBT-E. Sessions will only be conducted in an online setting. The content of the modules are presented in appendix A (in Dutch).

5.1 Screen-to-screen CBT-E

The non-experimental group will Cognitive Behavior Therapy-Enhanced (CBT-E). However, the Covid-19 pandemic requested adaptations to CBT-E (Termorshuizen et al., 2020). As in other specialized centers CBT-E is offered to patients on a screen-to-screen basis (Murphy et al., 2020; Waller et al., 2020). Additional advantage of screen-to-screen CBT-E is increased access for patient's to specialized eating disorder treatment (Abrahamsson et al., 2018).

CBT-E is an evidence based psychotherapy for eating disorders. Each session follows a fixed structure, with agenda setting, review of homework, weighing, explanation of rationale of each session, and assignment of homework. Each session has a time frame of 50 minutes, except the first session which lasts 90 minutes. In total 20 sessions will be conducted over 20 weeks. The first four weeks two sessions a week, followed by 10 sessions over 10 weeks and at last three bi-weekly sessions. All therapists are trained CBT-E specialists (Fairburn, 2008). Screen-to-screen CBT-E will not be blended with guided self-help CBT-E during the study,. Screen-to screen CBT-E is a 100% screen-to-screen treatment.

Step	Focus	Period (20 weeks in total)	Amount of sessions

Phase1, Step 1	Starting well	1 week	1
Step 2	Establish a regular eating pattern & Alternatives for binge eating	3 weeks	6
Phase 2, Step 3	Evaluation	1 week	2
Phase 3, Step 4	Module: shape concern	Distributed over 10 weeks	10
Step 4	Module: dietary restraint	Distributed over 10 weeks	10
Step 4	Module: Moods and events	Distributed over 10 weeks	10
Phase 4, Step 5	Ending well	6 weeks	3

5.1 Use of co-intervention (if applicable)

Not applicable

5.1 Escape medication (if applicable)

Not applicable

1. INVESTIGATIONAL PRODUCT

Not applicable

1. NON-INVESTIGATIONAL PRODUCT

Not applicable

2. METHODS

Study parameters/endpoints

Main study parameter/endpoint

Main parameter is to compare the efficacy regarding robust remission of guided self-help CBT-E compared with screen-to-screen CBT-E. Efficacy will be compared based on reduction in binge eating episodes and reduction in eating disorder pathology, measured by the EDE (Cooper & Fairburn, 1987) and EDE-Q (Fairburn & Beglin, 2008), together defining robust remission. Robust remission is defined as the portion of patients with a global score under one standard deviation above the community mean using international norms (EDE global score <1.74; EDE-Q global score <2.77), and no additional eating disorder behaviors (binging) (Jacobsen and Truax; EDE; EDEQ Fairburn).

Amount of objective binge eating episodes will be measured at start, during week 5 of treatment, , and at end of treatment (week 20 for screen-to-screen CBT-E and week 12 for guided self-help CBT-E) by the EDE (Cooper & Fairburn, 1987) and EDE-Q (Fairburn & Beglin, 2008). Follow up measures will be conducted because treatment effect is expected to continue after end of treatment (Fairburn et al., 2015; Fairburn et al., 2009). Follow up

measures in number of objective binge eating episodes will be collected by the EDE-Q (Fairburn & Beglin, 2008) 20 weeks post treatment and by the EDE (Cooper & Fairburn, 1987) 60 weeks post treatment. Due to time differences in end of treatment these measurements will be collected synchronous regarding treatment phase, but asynchronous in time, follow up 20 weeks post treatment (week 32 for guided self-help CBT-E, week 40 for screen-to-screen CBT-E) and 60 weeks (week 72 for guided self-help CBT-E, week 40 for screen-to-screen CBT-E) after treatment completion. Treatment is considered to be effective regarding reduction in binging once binge eating is reduced by 46%. This effect is comparable to treatments targeting binge eating behavior (Hilbert et al., 2019).

Eating disorder pathology, defined as restraint eating, dieting, weight concern and shape concern, will be measured at start, and at end of treatment (week 20 versus week 12) by the EDE (Cooper & Fairburn, 1987) and EDE-Q (Fairburn & Beglin, 2008). During week 5 of treatment the EDE-Q will be completed. Follow up measures will be conducted because treatment effect is expected to continue after end of treatment (Fairburn et al., 2015; Fairburn et al., 2009). Follow up measures regarding eating disorder pathology will be collected by the EDE-Q (Fairburn & Beglin, 2008) 20 weeks post treatment and by the EDE at 60 weeks post treatment (Cooper & Fairburn, 1987). Due to time differences in end of treatment these measurements will be collected synchronous regarding treatment phase, but asynchronous in time, follow up 20 weeks post treatment (week 32 for guided self-help CBT-E, week 40 for screen-to-screen CBT-E) and 60 weeks (week 72 for guided self-help CBT-E, week 40 for screen-to-screen CBT-E) after treatment completion. (see Figure 1 and Table 2). Treatment is considered to be effective regarding reduction in eating disorder pathology with a reliable change index (RCI) established as 0.67 change on the EDE and EDE-Q global score (Kazdin 2003; Kendall 1999).

Secondary study parameters/endpoints

Secondary outcome variables are improvements in quality of life measured by the five-level variant of the five-dimensional EuroQol instrument (EQ-5D) (Herdman et al 2011). A Dutch version for the EQ-5D-5L has been made available (Versteegh et al., 2016). Health care costs and productivity gains/losses will be measured using the TiC-P (Bouwmans et al., 2013). Furthermore, follow-up measurements are needed for the economic evaluation which will be performed alongside the RCT in line with ISPOR 2015 guidelines (Ramsey et al., 2015). Clinical impairment will be assessed through the Clinical Impairment Assessment (CIA) (Bohn et al., 2008). The EQ-5D-5L, TiC-P, and CIA will be completed at week 5 of treatment, and at end of treatment (week 20/ week 12). Due to time differences in end of treatment follow up and end of treatment measurements will be collected synchronous regarding treatment phase, but asynchronous in time, follow up 20 weeks post treatment (week 32 for guided self-help CBT-E, week 40 for screen-to-screen CBT-E) and 60 weeks (week 72 for guided self-help CBT-E, week 40 for screen-to-screen CBT-E) after treatment completion. (see Figure 1 and Table 2).

Secondary study parameters/endpoints (if applicable)

Secondary aim will be to assess if severity of body dissatisfaction and BMI at start moderate treatment outcome. In addition, age of onset of menarche will be assessed as a risk factor

body dissatisfaction. All factors will be assessed at start of treatment. Age of onset of menarche will be included in the assessment of demographic variables. BMI will be assessed by the EDE, as the EDE involves questions regarding weight and height (Cooper & Fairburn, 1987). Body dissatisfaction will be assessed by the body shape questionnaire (BSQ) (Cooper et al., 1987). In addition, reduction in body dissatisfaction will also be a treatment outcome variable and therefore the BSQ will also be completed at end of treatment and during follow-up: 20 weeks and 60 weeks after treatment completion (see Figure 1 and Table 2).

Other study parameters:

In addition, other variables will be collected such as: socio-demographic information (e.g. age, gender, domestic situation, occupational/student status, education level), they will be assessed as treatment moderators. Last, therapeutic alliance will be measured through the Working Alliance Inventory (WAI) (Hatcher & Gillaspy, 2006). The WAI will be completed at week 5, 12 and at end of treatment.

Table 2. Data collection instruments & timetable

Categories / outcome measure	Instrument (number of items)	Subcategories	Baseline	Week 5	End of treatment	20 weeks post treatment	60 weeks post treatment
Socio-demographic characteristics	Demographics (21)	Age, gender, domestic situation, ethnicity, occupational/ student status, education level, onset of menarche	x				
Days with binge eating episodes	Eating Disorder Examination-Questionnaire (EDE-Q) (28)	Shape concern<br>Weight concern<br>Restraint<br>Dieting	x	x	x	x	
	Eating Disorder Examination (EDE) (35)	Shape concern<br>Weight concern<br>Restraint<br>Dieting	x	x	x		x
Cost effectiveness	Questionnaire on Costs associated with Psychiatric illness (TiC-P) (40)		x		x	x	x
Quality of life	Quality of life questionnaire (EQ-5D-5L) (5)		x		x		
Therapeutic alliance	Working Alliance Inventory (WAI) (36)			x	x		

**Clinical Impairment**	Clinical Impairment Assessment (CIA) (16)	Personal Social Cognitive	x	x	x	x	x
**Body dissatisfactio n**	Body shape questionnaire (BSQ) (34)		x	x	x	x	x

Randomization, blinding and treatment allocation

Randomization takes place after the baseline assessment. Dual arm allocation will be performed in a 1:1 ratio using BMI ratio as allocator. BMI groups will be 19.5 < BMI< 35 and 35≤ BMI< 40. Randomization will be performed digitally by Castor EDC (https://www.castoredc.com/) by an independent data analyst provided by Arkin Mental Health care institute.

Study procedures

Recruitment, screening and informed consent procedure

Participants will be recruited at Novarum, the eating disorder treatment department of one of the largest mental health institutes in Amsterdam, Arkin. All patients referred to one of the participating sites are screened for eligibility by a clinician in an initial intake session. All eligible patients receive verbal and written study information during an advisory session. During this session participants will receive an informed consent, explaining research goals and information about participation in the study. Patients willing to participate in this study sign the informed consent form. It will be clearly indicated on this form that participation in the study is entirely voluntary and refusing to take part in the study is without any negative consequences. Patients who prefer not to participate will receive guided self- help CBT-E.

After the informed consent form is signed, baseline assessment takes place. An appointment will be scheduled in order to conduct the EDE by phone and a link will be sent to the participant in order to complete the questionnaires. The participant can complete the self- report questionnaires baseline assessment at home via computer/tablet/smartphone. Baseline assessment will be conducted through self- report questionnaires and an interview conducted by phone. All self-report assessments will be conducted via internet using CastorEDC which is ISO2700/27002/9001 and NEN 7510 certified.

After the baseline assessment has been completed, participants are randomized to either the experimental condition (guided self-help CBT-E) or the non- experimental condition (screen-to-screen CBT-E). Participants assigned to the experimental condition will start their treatment directly after they read the mandatory literature. The non- experimental group will start treatment after baseline assessment. Participants will receive an incentive of €20,- in gift cards after completion of 60 weeks follow-up measurement. Participants who refuse study participation will receive guided self-help CBT-E.

Follow- up assessments procedures

Treatment efficacy, robust remission defined as significant decrease in eating disorder pathology and no binge eating behaviors will be assessed starting from 20 weeks after end of treatment through self- report questionnaires. This will be at week 40 (screen-to-screen CBT-E) or week 32 (guided self-help CBT-E) , and week 80 (screen-to-screen CBT-E) or

week 72 (guided self-help CBT-E), for details see Table 2. All assessments will be conducted via internet using CastorEDC which is ISO2700/27002/9001 and NEN 7510 certified. Participants who do not complete an assessment will receive a reminder within one week via e-mail. If participants have not completed assessment after another week, they will be approached by phone by a research assistant. The research assistant will motivate them to complete the assessment and can guide them with possible assessment questions. Participants will receive an incentive of €20 in gift cards in total after completion of the measurements 60 weeks post treatment. See Table 2 for an overview of used instruments and assessment schedule.

Withdrawal of individual subjects

Subjects can leave the study at any time for any reason if they wish to do so without any consequences. The investigator can decide to withdraw a subject from the study for urgent medical reasons.

Specific criteria for withdrawal (if applicable)

In case a participant in both, the experimental and non- experimental condition meets the criteria of anorexia nervosa or bulimia nervosa and e.g. engages in self- induced vomiting the participant will be withdrawn from the study. This participant will continue face to face treatment instead. Another reason for study withdrawal will be development of suicidal ideations. In this case the participant will receive face to face treatment accompanied by psychopharmacologic treatment. Other reasons for study withdrawal will be meeting the criteria of another disorder as primary classification. In that case the participant will be referred to the appointed treatment site.

Replacement of individual subjects after withdrawal
Subjects who actively withdraw from the study will not be replaced.

Follow-up of subjects withdrawn from treatment
Subjects withdrawn from the study will not be assessed during follow up. In addition, withdrawn participants can request their data to be excluded from analysis within this study. Participants who showed non-compliance regarding follow-up measures will be assessed according to an intention-to-treat analysis. Missing data will be handled by using multiple imputation methods.

Premature termination of the study
Other than adverse events, there are no criteria for a premature termination of the study

3. SAFETY REPORTING

Temporary halt for reasons of subject safety

In accordance to section 10, subsection 4, of the WMO, the sponsor (Novarum center for eating disorders) will suspend the study if there is sufficient ground that continuation of

the study will jeopardize subject health or safety. The sponsor will notify the accredited METC without undue delay of a temporary halt including the reason for such an action. The study will be suspended pending a further positive decision by the accredited METC. The investigator will take care that all subjects are kept informed.

AEs, SAEs and SUSARs

Adverse events (AEs)

Adverse events are defined as any undesirable experience occurring to a subject during the study, whether or not considered related to [the investigational product / trial procedure/ the experimental intervention]. All adverse events reported spontaneously by the subject or observed by the investigator or his staff will be recorded.

Serious adverse events (SAEs)

A serious adverse event is any untoward medical occurrence or effect that

- results in death;
- is life threatening (at the time of the event);
- requires hospitalization or prolongation of existing inpatients' hospitalization;
- results in persistent or significant disability or incapacity;
- is a congenital anomaly or birth defect; or
- any other important medical event that did not result in any of the outcomes listed above due to medical or surgical intervention but could have been based upon appropriate judgement by the investigator.

An elective hospital admission will not be considered as a serious adverse event.

The investigator will report all SAEs to the sponsor without undue delay after obtaining knowledge of the events. The investigator will report all SAEs to the sponsor without undue delay after obtaining knowledge of the events. The sponsor will report the SAEs through the web portal *ToetsingOnline* to the accredited METC that approved the protocol, within 7 days of first knowledge for SAEs that result in death or are life threatening followed by a period of maximum of 8 days to complete the initial preliminary report. All other SAEs will be reported within a period of maximum 15 days after the sponsor has first knowledge of the serious adverse events.

Suspected unexpected serious adverse reactions (SUSARs)

Not applicable

Annual safety report

Not applicable

Follow-up of adverse events

All AEs will be followed until they have abated, or until a stable situation has been reached. Depending on the event, follow up may require additional tests or medical procedures as indicated, and/or referral to the general physician or a medical specialist.

SAEs need to be reported till end of study within the Netherlands, as defined in the protocol

[Data Safety Monitoring Board (DSMB) / Safety Committee]

A data safety monitoring board is not established for this study.

The advice(s) of the DSMB will only be sent to the sponsor of the study. Should the sponsor decide not to fully implement the advice of the DSMB, the sponsor will send the advice to the reviewing METC, including a note to substantiate why (part of) the advice of the DSMB will not be followed.

4. STATISTICAL ANALYSIS

Primary study parameter(s)

SPSS version 25 will be used for statistical analysis. Whether baseline scores and demographics (age, gender, level of education, eating disorder severity and clinical characteristics) predict treatment completion will be examined by chi-square analyses for categorical variables and a binomial logistic regression for dimensional variables. Significance of baseline differences between the guided self-help CBT-E and screen-to-screen CBT-E groups will be examined by a Chi-square or *t*-test. Reduction in eating disorder pathology from start to evaluation and EOT, reported as means (standard deviation), will be measured by a pairwise repeated measures ANOVA. Effect sizes will be calculated using Cohen's *d* (0.2 small, 0.5 medium, 0.8 large) (Cohen 1977) and adjusted for bias (Hedges 1981). Linear mixed model analyses with fixed effect at the individual level will be performed to examine the effect treatment group on robust remission at start, evaluation, EOT and follow up measures. During treatment the EDE and EDE-Q will be used to define robust remission rate (reported as %), during follow-up 20 weeks only the EDE-Q and during follow-up 60 weeks only the EDE. The groups will be nested in their treatment group (guided self-help or screen-to-screen) and fixed effects are age and gender. The interaction between robust remission rate, treatment condition and the fixed effects will be measured as well. Time will be defined as the time of treatment and follow-up. The contrasts chosen compared guided self-help to the screen-to-screen group at start, evaluation (week 5), EOT and follow up 20, and 60 weeks. Analyses will primarily be performed according to an intention-to-treat (ITT) approach (imputed dataset with 50 imputations for each missing observation), which includes all patients and additionally and on completers (complete measures available at baseline, evaluation, EOT and follow up). Multiple imputation will be used for missing outcome data, under a missing-at-random (MAR) assumption. First, analyses will be performed on the imputed datasets separately. Next, the outcomes of the 50 imputations were combined using Rubin's rules (Rubin, 1987).

Secondary study parameter(s)

Quality of life and clinical impairment will be reported as means (standard deviations); and effect sizes between the two conditions will be reported as Cohen's d. Direct costs of the eating disorder treatment will be reported in euros. Cost effectiveness analysis with robust remission as effect measure and a cost- utility analysis using QALYs will be performed. The QALYs will be derived from the Dutch version of EQ-5D-5NL (Hakkaart-van Roijen, Straten, Tiemens, & Donker, 2002). Generalized Linear Mixed Models will be used for the analysis of intervention outcomes. Analyses will be conducted on the entire randomized sample (i.e. intention to treat), and on the per protocol/ treatment completers sample. The primary endpoint for the study is end of treatment (12 or 20 weeks post randomization). As there are no multiple primary endpoints, there will be no Bonferroni correction or other correction for the significance level applied. Missing data will be handled using multiple imputation.

Secondary study parameter(s)

Multiple regression analyses will be performed in order to determine treatment outcome moderators (demographics, therapeutic alliance, severity of body dissatisfaction, and BMI). De moderators will be reported as means (standard deviations); and effect sizes between the two conditions will be reported as Cohen's d. To enhance power, the moderators will not only be investigated on robust remission but also on the continuous outcome variable of eating disorder pathology severity as measured by the EDE and EDE-Q. Outliers will be removed.

In order to establish the covariating effect of age of onset of menarche SPSS process package will be used. Age of onset will be will be reported as means (standard deviations); and effect sizes between the two conditions will be reported as Cohen's d. Outliers will be removed.

Study parameters

There are several study parameters, as described below

Eating disorder pathology

EDE The Eating Disorder Examination (EDE) (Cooper & Fairburn, 1987) is a semi-structured classification interview assessing eating disorder pathology during the last 28 days up to the last 6 months, including binge eating behavior. Eating disorder symptoms are measured on a 7 point Likert scale (Cooper & Fairburn, 1987). The EDE, involves of four subscales: dietary restraint, eating concern, weight concern and shape concern. The EDE, considered to be the golden standard for eating disorder assessment, has good internal consistency (Cooper et al., 1989), discriminative and concurrent validity (Rosen, Vara, Wendt, & Leitenberg, 1990), and test-retest reliability (Rizvi, Peterson, Crow, & Agras, 2000). The Dutch version of the EDE is validated by Jansen (2000) (Jansen, Fairburn, & Cooper, 2000).

EDE-Q The Dutch version of the Eating Disorders Examination-Questionnaire 6.0 (EDE-Q) (Aardoom, Dingemans, Slof Op't Landt, & Van Furth, 2012; Fairburn & Beglin, 2008) will be used to asses eating disorder pathology. Like the EDE, eating pathology will be measured on a 7 point Likert scale (Fairburn & Beglin, 2008). The Dutch version of the EDE-Q, has good psychometric properties (Aardoom et al., 2012). The EDE-Q is a self-report

questionnaire of 36 items, measuring bingeing behavior during the last 4 weeks (Fairburn & Beglin, 2008). The EDE-Q, involves of four subscales: dietary restraint, eating concern, weight concern and shape concern, however the four factor structure of the English original did not provide fit, therefore analysis will only be performed on EDE-Q global score.

Socio- demographic characteristics

Several demographic characteristics such as age, gender, marital status, domestic situation, level of education and occupational/ student status will be asked to the participants. These socio- demographic characteristics will serve as a moderator. Baseline differences in demographic characteristics will be analyzed using X^2 tests and *t*-tests. Effects will be calculated through a regression analysis and reported as beta (β), including *p* value.

Body dissatisfaction

The Body Shape Questionnaire (BSQ) (Cooper et al., 1987) is a widely used self-report measure to assess body dissatisfaction. It consists of 34 questions to be answered on a 6 point Likert scale (never, rarely, sometimes, often, very often and always). Good psychometric properties such as concurrent and discriminant validity, internal consistency and test-retest reliability have been attested in many studies and in different languages (Lentillon-Kaestner, Berchtold, Rousseau, & Ferrand, 2014).

Age of onset of menarche

It has been suggested that early pubertal development may foster body image and eating disturbances; as early menarche leads to increased adipose tissue, it moves girls away from the normative body shape of adolescents which theoretically increases body dissatisfaction and consequent dieting and eating disturbances. Early menarche might therefore serve as a covariate Findings however are inconclusive for the claim that early menarche is a risk factor for body dissatisfaction and eating pathology (Mitchison & Hay, 2014; Stice, 2002). These inconclusiveness may be related to the fact that the adverse effects of early menarche are developmentally localized and may dissipate over time (Stice, 2002). Participants will be asked their age of onset of menarche and will be asked whether they experienced developmental deviance at time of onset.

Therapeutic alliance

Therapeutic alliance improves treatment effectiveness (Abrahamsson et al., 2018; Fernandez, Salem, Swift, & Ramtahal, 2015). Therapeutic alliance will be measured through the Working Alliance Inventory (WAI). The WAI is a 36- item self- report questionnaire addressing working alliance between patient and therapist. However, only the patient's perspective will be assessed in this study. The 36 items are scored on a 7 point Likert scale, resulting in 3 dimensions, namely Bonds, Tasks and Goals (Hatcher & Gillaspy, 2006). The Dutch version is validated by Paap (2019). These continuous data have a quantitative nature. Missing data will be handled using multiple imputation in the software package R 3.0+ or SPSS. Analyses will be conducted on the entire randomized sample (i.e. intention to treat) and on the per protocol/ treatment completers sample. All analyses will be carried out using SPSS version 25+ and/or R version 3.0+. Outcomes will be displayed as means

(standard deviations). Effects will be calculated through a regression analysis and reported as beta (b), including *p* value.

Clinical impairment assessment

Eating disorders come along with several psychosocial impairments due to eating and compensatory behavior, as well as their concerns regarding shape, weight and eating pattern. The Clinical Impairment Assessment (CIA) differentiates between eating disorder psychopathology and the impairment secondary to eating disorder psychopathology (Bohn et al., 2008). The CIA is a 16- item self report questionnaire. Items are rated on a 4 point Likert scale. Clinical impairment is assessed across specific areas and consists of three subscales, namely personal, social and cognitive domains (Reas, Ro, Kapstad, & Lask, 2010). The Dutch version of the CIA is validated by Schlochtermeier (2021) (Schlochtermeier, 2021). The continuous data have a quantitative nature. Missing data will be handled using multiple imputation in the software package R 3.0+ or SPSS. Analyses will be conducted on the entire randomized sample (i.e. intention to treat) and on the per protocol/ treatment completers sample. All analyses will be carried out using SPSS version 25+ and/or R version 3.0+. Outcomes will be displayed as means (standard deviations). Effects will be calculated through a regression analysis and reported as beta (b), including *p* value.

Economic evaluation

Costs

Several types of costs will be assessed: (1) the costs of offering the intervention, (2) patients' out-of-pocket expenses, (3) costs stemming from productivity losses due to absenteeism or reduced efficiency while at work (presenteeism). The first type of costs is also known as the direct medical costs and will be based on the full economic costs of offering the interventions. This study will apply the Dutch guideline for economic evaluation (Zorginstituut, 2016), and rely on the standard cost prices reported. In addition, the participants electronic file will be searched to make additional calculations on costs of guided self-help CBT-E versus screen-to-screen CBT-E. Productivity losses will be based on the gender- and age-specific friction costs. Data on resource use (health care uptake) and productivity losses will be collected with a Dutch version of the widely used TiC-P (Hakkaart-van Roijen et al., 2002).

Effects

As effect measure for the cost-effectiveness analysis, the Treatment Response primary outcome variable will be used. For the cost-utility analyses Dutch version EQ-5D-5L (Versteegh et al., 2016) will be used to compute health gains expressed in quality adjusted life years (QALYs). Missing data will be handled using multiple imputation in the software package R 3.0+ or SPSS. Analyses will be conducted on the entire randomized sample (i.e. intention to treat) and on the per protocol/ treatment completers sample. All analyses will be carried out using SPSS version 25+ and/or R version 3.0+. Outcomes will be displayed as means (standard deviations). Effects will be calculated through a regression analysis and reported as beta (b), including *p* value.

Cost-effectiveness calculations

The economic evaluation will be conducted alongside the randomized trial. The Dutch tariffs (utility weights) (Versteegh et al., 2016) for the EQ-5D will be used for computing the QALYs; for the MOS SF-36, the Brazier scoring algorithm (SF-6D) will be used (Brazier et al 2002). Using the area under the curve (AUC) method, the periods between the measurement waves will be weighted by the utility of the health state in that period. This allows the computation of quality adjusted life years (QALYs) over the entire trial period. In similar vein, cumulative costs over the entire follow-up period will be obtained from the cost estimates at the various measurement waves. The cost-effectiveness evaluation will be performed in line with suggestions by Drummond et al. (2015), i.e. in agreement with the intention-to-treat principle, with missing data addressed using imputation. The incremental cost-effectiveness ratio (ICER) will be calculated as follows: ICER = (C1 – C2)/(E1 –E2), where Care costs, E effects, and subscripts 1 and 2 refer to the two trial arms (screen-to-screen or guided self-help CBT-E). Confidence intervals around the ICER will be calculated using a non-parametric bootstrap approach: 2,500 non-parametric bootstrapped samples will be extracted from each of the original datasets. For each of these bootstrapped samples, the incremental costs, incremental effects, and the incremental cost-effectiveness ratio (ICER) will be calculated. The resulting 2,500 ICERs per dataset will be used for further calculations and will be plotted on a cost-effectiveness plane. In addition, cost-effectiveness acceptability curves (CEACs) will plotted. One-way sensitivity analyses directed at uncertainty in the main cost drivers will be performed to gauge the robustness of our findings.

Interim analysis (if applicable)

Not applicable

5. ETHICAL CONSIDERATIONS

Regulation statement

This study will be conducted according to the principles of the Declaration of Helsinki (64th WMA General Assembly, Fortaleza, Brazil, October 2013) and in accordance with the Medical Research Involving Human Subjects ACT (Dutch abbreviation: WMO).

Recruitment and consent

Participants will be recruited at Novarum, the eating disorder treatment department of Arkin, one of the largest mental health institutes in Amsterdam. All patients referred to this site are screened for eligibility by a clinician during a regular initial intake session. The clinician conducting the initial intake session will be the patients primary contact person and will be authorized to report and verify medical information into the patient's personal medical file. All eligible patients receive written study information during an advisory session, in general 1-2 weeks after the initial intake session was conducted. Patients who agree to participate will sign an informed consent during this session. The informed consent explains the research goals, provides information about participation in the study. Also, the patient has the possibility to ask additional questions. In case the patient is not convinced to participate immediately, he/she has a maximum of 2 weeks to decide if he/she wants to participate in the study. Then, the research assistant makes either an appointment for signing the informed consent form, or the patient can send the signed informed consent form

to a research assistant, project leader or primary contact person if preferred. Once the informed consent form is signed and well received the research assistant will schedule a phone call appointment in order to conduct baseline assessment and the research assistant will send a link to the participant in order to conduct the self report questionnaires of baseline assessment.

Participants will be given the opportunity to ask questions regarding the study or participation. Contact information (phone/e-mail) will be given, so that participants can contact a member of the research team when they have questions concerning the study. Patients willing to participate in this study sign the informed consent form. It will be clearly indicated on this form that participation in the study is entirely voluntary and refusing to take part in the study is without any negative consequences. Furthermore, it will be stressed that participants can redraw their participation in the study at any time, without given a reason why they wish to stop participating. Only after the informed consent form is signed, the baseline assessment takes place. See attachments in folder E for patient information letter and informed consent form.

Objection by minors or incapacitated subjects (if applicable)
Participants need to be 18 years old or older to participate in the study. Participants who are unable to give their informed consent will not be included.

Benefits and risks assessment, group relatedness
The expected risk concerning participation in this study are expected to be negligible. Possible risk could entail mild feelings of restlessness such as an urge to binge. Furthermore, participants are asked to complete the follow-up questionnaires and/or keep an diary regarding their eating habits. Therefore, they might could be more aware of their eating behavior in case of set-back.

Compensation for injury
The sponsor/investigator has a liability insurance which is in accordance with article 7 of the WMO. The sponsor prefers to obtain dispensation from the statutory obligation to provide insurance, because risk level of participating in this study is negligible. A dispensation request has been made to the accredited METC. When the request is granted, it will be reported in this section of the next version of this proposal.

Incentives
Participants will receive a total remuneration of €20,- in gift cards for their participation in the study, including completion of the follow-up measurement wave (20, and 60 weeks after end of treatment).

6. ADMINISTRATIVE ASPECTS, MONITORING AND PUBLICATION

Handling and storage of data and documents
When included in the study, all participants will be given an unique project number (de-identification). Key of these project numbers will only be available to the relevant project data managers, not including the investigators. All questionnaires and data will be stored and handled using this project number. Study outcomes will be reported anonymously. Data will

be stored within the European Union and complies with the European General Data Protection Regulation (in Dutch Algemene Verordening Gegevensbescherming, AVG). Storage of data will be supervised by the principal investigator. Arkins experienced research data management departments will be involved in formalization of the data management plan in accordance with ISO 27001/27002/9001 and the Dutch NEN 7510 norm for information security in healthcare.

Karify will be used to develop guided self-help CBT-E (see http://my.karify.com). Programs and user data from this platform are hosted on a cloud in Europe from a provider which adheres to common security standards such as ISO 27001 and is NEN 7510 certified. ISO 27001 is a specification for an information security management system: a framework of policies and procedures that includes all legal, physical and technical controls involved in an organization's information risk management processes.

Contact between patient and therapist during both, guided self-help CBT-E and screen-to-screen CBT-E will be offered through MSteams, which is ISO 27002 and BSI C5 certified.

Monitoring and Quality Assurance

Both, guided self-help CBT-E and screen-to-screen CBT-E are based on the CBT-E treatment protocol provided by Christopher Fairburn (Fairburn, 2008). In addition, study quality will be assured by following Arkin quality standards. Researchers might be audited during the study duration. During an audit an independent researcher will investigate whether the study is being or has been done based on the quality standards of Arkin.

Amendments

Amendments are changes made to the research after a favourable opinion by the accredited METC has been given. All amendments will be notified to the METC that gave a favourable opinion. All substantial amendments will be notified to the METC and to the competent authority. Non-substantial amendments will not be notified to the accredited METC and the competent authority, but will be recorded and filed by the sponsor.

Annual progress report

The sponsor/investigator will submit a summary of the progress of the trial to the accredited METC once a year. Information will be provided on the date of inclusion of the first subject, numbers of subjects included and numbers of subjects that have completed the trial, serious adverse events/ serious adverse reactions, other problems, and amendments.

Temporary halt and (prematurely) end of study report

The investigator/sponsor will notify the accredited METC of the end of the study within a period of 8 weeks. The end of the study is defined as the last patient's last visit.
The sponsor will notify the METC immediately of a temporary halt of the study, including the reason of such an action. In case the study is ended prematurely, the sponsor will notify the accredited METC within 15 days, including the reasons for the premature termination.

Within one year after the end of the study, the investigator/sponsor will submit a final study report with the results of the study, including any publications/abstracts of the study, to the accredited METC.

12.6 Public disclosure and publication policy

As all data is only fully available to the Sponsor there is no need for agreements on publication restrictions among involved parties. Results will be written in a journal article and submitted for publication. Authorship will be handled according to usual conventions Participants will be informed via a newsletter about the results if they had requested so at registration.

If the interventions prove to be as successful in reducing binge eating episodes among participants as we anticipate based on previous findings, an implementation plan will be developed and executed in collaboration with patient organisations.

Outcomes and experiences will be presented to and shared with relevant stakeholders such as: (a) (international) clinicians working in eating disorder care: (b) (international) researchers: (c) participants working on the development of Dutch Multidisciplinary Guidelines for eating disorder treatment treatment (eg. Nederlandse Academie voor Eetstoornissen, Nederlands Instituut van Psychologen); (d) Dutch eating disorder patient organizations, e.g. stichting WEET, stichting JIJ, Proud2bme (e) Insurance companies and policymakers with regard to mental health care. In addition, ...

In addition, publications in peer reviewed journals, presentations, and other evidence-based information will be shared at Novarum, center for eating disorders' website (www.novarum.nl) and project facebook page (https://www.facebook.com/BEDonlineNovarum). News updates regarding achieved milestones of this project will be published on these websites and will be shared through social media channels and newsletters of participating organizations. Information on this project will also be published on websites frequently visited by mental health care patients. Studies involving this research project will be submitted to (inter)national peer-reviewed journals.

7. STRUCTURED RISK ANALYSIS

CE tool (https://cetool.nl/medisch-hulpmiddel/) was applied to conduct a risk analysis, both guided self-help CBT-E and screen-to-screen CBT-E are classified as a class 1 medical device. This means that both treatment involve a low risk for participants. Karify is responsible to provide certification of the medical device. Starting from 26 May 2021 CE certification will be mandatory by Dutch law (see appendix in folder D IMDD). Karify is currently processing such certification and will make sure the process is finalized by May 26th.

8. REFERENCES

Aardoom, J. J., Dingemans, A. E., Slof Op't Landt, M. C., & Van Furth, E. F. (2012). Norms and discriminative validity of the Eating Disorder Examination Questionnaire (EDE-Q). *Eat Behav, 13*(4), 305-309. doi:10.1016/j.eatbeh.2012.09.002

Abdullah, S., Matthews, M., Frank, E., Doherty, G., Gay, G., & Choudhury, T. (2016). Automatic detection of social rhythms in bipolar disorder. *J Am Med Inform Assoc, 23*(3), 538-543. doi:10.1093/jamia/ocv200

Abrahamsson, N., Ahlund, L., Ahrin, E., & Alfonsson, S. (2018). Video-based CBT-E improves eating patterns in obese patients with eating disorder: A single case multiple baseline study. *J Behav Ther Exp Psychiatry, 61*, 104-112. doi:10.1016/j.jbtep.2018.06.010

Arnberg, F. K., Linton, S. J., Hultcrantz, M., Heintz, E., Jonsson, U., & Davey, C. G. (2014). Internet-Delivered Psychological Treatments for Mood and Anxiety Disorders: A Systematic Review of Their Efficacy, Safety, and Cost-Effectiveness. *PloS one, 9*(5), e98118. doi:10.1371/journal.pone.0098118

Becker, A. E., Hadley Arrindell, A., Perloe, A., Fay, K., & Striegel-Moore, R. H. (2010). A qualitative study of perceived social barriers to care for eating disorders: perspectives from ethnically diverse health care consumers. *Int J Eat Disord, 43*(7), 633-647. doi:10.1002/eat.20755

Berg, E., Schlochtermeier, D., Koenders, J., Mooij, L., Goudriaan, A., Blankers, M., . . . Dekker, J. (2020). Implementing cognitive behavioral therapy-enhanced in a routine inpatient and outpatient setting: Comparing effectiveness and treatment costs in two consecutive cohorts. *International Journal of eating disorders, 53*(3), 461-471. doi:10.1002/eat.23229

Bohn, K., Doll, H. A., Cooper, Z., O'Connor, M., Palmer, R. L., Fairburn, C. G. J. B. r., & therapy. (2008). The measurement of impairment due to eating disorder psychopathology. *46*(10), 1105-1110. Retrieved from https://www.sciencedirect.com/science/article/pii/S0005796708001265?via%3Dihub
https://pdf.sciencedirectassets.com/271799/1-s2.0-S0005796708X00105/1-s2.0-
S0005796708001265/main.pdf?X-Amz-Security-
Token=AgoJb3JpZ2luX2VjEDkaCXVzLWVhc3QtMSJHMEUCIQCotDge384%2FsAd6
UoemgXA1%2BkNSl%2FxCkgcVlk4b2QkmuAIgCEM%2FhNczYwkqMtGtMS9cpwSY
vws7R5TT4j%2BL9wlc1FMq4wMIwv%2F%2F%2F%2F%2F%2F%2F%2F%2F%2FA
RACGgwwNTkwMDM1NDY4NjUiDLHWyuik6OpWfpwP%2Fyq3A5k3CMXzsbcuZ0Xe
KXtmfvVmTNeU29iEuGM3Jy8Vr5DYjdGmnWXpUFdKkJw6ZP2JGwNhCfZXQ55TkiG
OXtTxIcMzhM%2BJ8meTH5UUMfi6Baf142L4QBR6qA%2FK9svSHc6fxqwzaXxbNu7
RvPIe%2FVawHcp9bHB9IWiA%2Be%2FdzlFyFLJeI3VN4NBA11dRWc4QxwrxHse5e
mO4AkQy9%2F56Ir9YBa6Fpg%2FnfObREWgn2fhF1XaBQa75NtYnXcFziCVR8SWp
LOBskoupBCgVJqyPg7yfStXitZcfoOBi2v38i1prz3wcEyDEQIWXNJ%2BYIE4kXau7%
2FXZqY4crOoAff4O5sUOKNII0K4ExnfQy%2FUd0G032Nzi%2FVulbLvvDZCGprB8E
3lQphmAtCZ4uxPYKWieB7k3ARR635WQ1OVmq6bx9I6pzCCE716UgZozpJmimixhV
IhUBgTK2hIQTDCi%2BrbNmuswhlGByfWwO0cc%2BYnWcrFBhZFwBQoX2DCci6N
V8mIMo3KV97AXJPFIMzczrZZwgRRFxhSMEeFHsPec98dcIrRvc28YtCShr05pVwx4
5z7QzexSW5eR4cCuA%2Fflw0InB6gU6tAHfX5%2FENa%2Fwu540TEMEdikHTFZG
Sye0PK8sAgiCXAiN6RpSrzjehAxmffzhf6%2BNivEyUzqAc4bstBZyn%2FX2JYx%2Bh
SWnLonmJFwRmAzI3oeKU1CaLHCwTi36WZWCkRDQ9pYHMZJr%2BaPvmmLWB
W5NmoYymbL1V3CVpg3SU3QUiCHa9VrK2OFhadnCG1RxDZmMmK6FDoKFohteH
bnl06zYopREdnAdpnnK%2BjzpGt4t7yimo6OxJGk%3D&X-Amz-Algorithm=AWS4-
HMAC-SHA256&X-Amz-Date=20190811T173144Z&X-Amz-SignedHeaders=host&X-
Amz-Expires=300&X-Amz-
Credential=ASIAQ3PHCVTY6FPIPWNY%2F20190811%2Fus-east-
1%2Fs3%2Faws4_request&X-Amz-
Signature=1b6f650b9a1e2a1d95801241451386b93f198e0142b003ef7b7d1c8df5f365

17&hash=6785ea107e5579222957092c5335d160daab6af20201ab4e31bb2a54875072a6&host=68042c943591013ac2b2430a89b270f6af2c76d8dfd086a07176afe7c76c2c61&pii=S0005796708001265&tid=spdf-949d86e8-f689-46a4-81b0-e4afafba38ee&sid=e74e33891335f749b87a462522255d6e729fgxrqb&type=client

Bouwmans, C., De Jong, K., Timman, R., Zijlstra-Vlasveld, M., Van der Feltz-Cornelis, C., Tan Swan, S., & Hakkaart-van Roijen, L. (2013). Feasibility, reliability and validity of a questionnaire on healthcare consumption and productivity loss in patients with a psychiatric disorder (TiC-P). *BMC Health Serv Res, 13*(1), 217. doi:10.1186/1472-6963-13-217

Bulik, C. M., Marcus, M. D., Zerwas, S., Levine, M. D., & La Via, M. (2012). The Changing "Weightscape" of Bulimia Nervosa. *American Journal of Psychiatry, 169*(10), 1031-1036. doi:10.1176/appi.ajp.2012.12010147

Cooper, P. J., Taylor, M. J., Cooper, Z., & Fairbum, C. G. J. I. J. o. e. d. (1987). The development and validation of the Body Shape Questionnaire. *6*(4), 485-494.

Cooper, Z., Cooper, P. J., & Fairburn, C. G. (1989). The validity of the eating disorder examination and its subscales. *Br J Psychiatry, 154*(6), 807-812. doi:10.1192/bjp.154.6.807

Cooper, Z., & Fairburn, C. (1987). The eating disorder examination: A semi-structured interview for the assessment of the specific psychopathology of eating disorders. *International Journal of eating disorders, 6*(1), 1-8.

de Zwaan, M., Herpertz, S., Zipfel, S., Svaldi, J., Friederich, H. C., Schmidt, F., . . . Hilbert, A. (2017). Effect of Internet-Based Guided Self-help vs Individual Face-to-Face Treatment on Full or Subsyndromal Binge Eating Disorder in Overweight or Obese Patients: The INTERBED Randomized Clinical Trial. *JAMA psychiatry, 74*(10), 987-995. doi:10.1001/jamapsychiatry.2017.2150

Evans, E. J., Hay, P. J., Mond, J., Paxton, S. J., Quirk, F., Rodgers, B., . . . Sawoniewska, M. A. (2011). Barriers to help-seeking in young women with eating disorders: a qualitative exploration in a longitudinal community survey. *Eat Disord, 19*(3), 270-285. doi:10.1080/10640266.2011.566152

Fairburn, C. (2008). *Cognitive behavior therapy and eating disorders.* New York: Guilford Press.

Fairburn, C., & Beglin, S. J. (1994). Assessment of eating disorders: interview or self-report questionnaire? *Int J Eat Disord, 16*(4), 363-370. doi:https://doi.org/10.1002/1098-108X(199412)16:4<363::AID-EAT2260160405>3.0.CO;2-%23

Fairburn, C. G. (2013). *Overcoming binge eating*: Guilford Press.

Fairburn, C. G. (2016). *Overwin je eetbuien, waarom je te veel eet en hoe je daarmee kunt stoppen* (L. Berkhuizen, Trans.). Amsterdam: Uitgeverij Nieuwezijds.

Fairburn, C. G., & Beglin, S. J. (2008). Eating Disorder Examination- Questionnaire (6.0).

Fairburn, C. G., Cooper, Z., & Shafran, R. (2003). Cognitive behaviour therapy for eating disorders: A "transdiagnostic" theory and treatment. *Behaviour research and therapy, 41*(5), 509-528. doi:10.1016/s0005-7967(02)00088-8

Fairburn, C. G., & Harrison, P. J. (2003). Eating Disorders. *Lancet, 361*, 407-416. Retrieved from https://www.thelancet.com/journals/lancet/article/PIIS0140-6736(03)12378-1/fulltext

Fairburn, C. G., & Peveler, R. C. (1990). Bulimia nervosa and a stepped care approach to management. *Gut, 31*(11), 1220-1222.

Fernandez, E., Salem, D., Swift, J. K., & Ramtahal, N. (2015). Meta-analysis of dropout from cognitive behavioral therapy: Magnitude, timing, and moderators. *J Consult Clin Psychol, 83*(6), 1108-1122. doi:10.1037/ccp0000044

Grilo, C. M. (2017). Psychological and Behavioral Treatments for Binge-Eating Disorder. *The Journal of clinical psychiatry, 78*(Suppl 1), 20-24. doi:10.4088/JCP.sh16003su1c.04

Grilo, C. M., Masheb, R. M., & Crosby, R. D. (2012). Predictors and Moderators of Response to Cognitive Behavioral Therapy and Medication for the Treatment of Binge Eating Disorder. *Journal of consulting and clinical psychology, 80*(5), 897-906.

Hakkaart-van Roijen, L., Straten, A. v., Tiemens, B., & Donker, M. (2002). *Handleiding Trimbos/iMTA questionnaire for Costs associated with Psychiatric illness (TiC-P)*. Retrieved from

Hatcher, R. L., & Gillaspy, J. A. J. P. R. (2006). Development and validation of a revised short version of the Working Alliance Inventory. *16*(1), 12-25.

Jansen, A., Fairburn, C. G., & Cooper, Z. (2000). *Eating disorder examination (EDE 12.0) : interview ter vaststelling van de specifieke psychopathologie van eetstoornissen*. Lisse: Swets Test Publishers (STP).

Kraemer, H. C. (2013). Discovering, comparing, and combining moderators of treatment on outcome after randomized clinical trials: a parametric approach. *Statistics in medicine, 32*(11), 1964-1973. doi:10.1002/sim.5734

Lentillon-Kaestner, V., Berchtold, A., Rousseau, A., & Ferrand, C. (2014). Validity and reliability of the French versions of the body shape questionnaire. *Journal of personality assessment, 96*(4), 471-477. doi:10.1080/00223891.2013.843537

Lynch, F. L., Striegel-Moore, R. H., Dickerson, J. F., Perrin, N., Debar, L., Wilson, G. T., & Kraemer, H. C. (2010). Cost-effectiveness of guided self-help treatment for recurrent binge eating. *J Consult Clin Psychol, 78*(3), 322-333. doi:10.1037/a0018982

Melisse, B., Berg, E. v. d., Jonge, M. d., Blankers, M., Dekker, J., & Beurs, E. d. (2021). *Online cognitive behavioral therapy-enhanced for binge eating disorder: a randomized controlled trial.*

Melisse, B., Jonge, M. d., Berg, E. v. d., Koenders, J., Peen, J., Dekker, J., & Beurs, E. d. (2021). Comparing the effectiveness of Cognitive Behavioral Therapy-Enhanced between patients with different Eating Disorder classifications: a naturalistic study.

Mitchell, J. E., Crosby, R. D., Wonderlich, S. A., Crow, S., Lancaster, K., Simonich, H., . . . Myers, T. C. (2008). A randomized trial comparing the efficacy of cognitive–behavioral therapy for bulimia nervosa delivered via telemedicine versus face-to-face☆. *Behaviour research and therapy, 46*(5), 581-592. doi:10.1016/j.brat.2008.02.004

Mitchison, D., & Hay, P. J. (2014). The epidemiology of eating disorders: genetic, environmental, and societal factors. *Clinical epidemiology, 6*, 89-97. doi:10.2147/CLEP.S40841

Murphy, R., Calugi, S., Cooper, Z., & Dalle Grave, R. (2020). Challenges and opportunities for enhanced cognitive behaviour therapy (CBT-E) in light of COVID-19. *The Cognitive Behaviour Therapist, 13*. doi:10.1017/S1754470X20000161

Myers, K., & Turvey, C. (2013). *Telemental health : clinical, technical, and administrative foundations for evidence-based practice* (1st ed. ed.). Amsterdam: Elsevier.

Paxton, S. J., McLean, S. n. A., Gollings, E. K., Faulkner, C., & Wertheim, E. H. (2007). Comparison of face-to-face and internet interventions for body image and eating problems in adult women: An RCT. *International Journal of eating disorders, 40*(8), 692-704. doi:10.1002/eat.20446

Ramsey, S. D., Willke, R. J., Glick, H., Reed, S. D., Augustovski, F., Jonsson, B., . . . Sullivan, S. D. J. V. i. H. (2015). Cost-effectiveness analysis alongside clinical trials II—an ISPOR Good Research Practices Task Force report. *Value Health, 18*(2), 161-172. doi:10.1016/j.jval.2015.02.001

Reas, D. L., Ro, O., Kapstad, H., & Lask, B. (2010). Psychometric properties of the clinical impairment assessment: norms for young adult women. *Int J Eat Disord, 43*(1), 72-76. doi:10.1002/eat.20653

Rizvi, S. L., Peterson, C. B., Crow, S. J., & Agras, W. S. (2000). Test-retest reliability of the eating disorder examination. *Int J Eat Disord, 28*(3), 311-316. doi:10.1002/1098-108x(200011)28:3<311::aid-eat8>3.0.co;2-k

Rosen, J. C., Vara, L., Wendt, S., & Leitenberg, H. J. I. J. o. E. D. (1990). Validity studies of the eating disorder examination. *9*(5), 519-528. doi:https://doi.org/10.1002/1098-108X(199009)9:5%3C519::AID-EAT2260090507%3E3.0.CO;2-K

Schlochtermeier, D., van den Berg, E., Koenders, K., Peen, J., Dekker, J. (2021). *Clinical Impairment Assessment: Psychometric properties of the Dutch version and norms for female clinical and non-clinical population*. Unpublished work.

Shafran, R., Clark, D. M., Fairburn, C. G., Arntz, A., Barlow, D. H., Ehlers, A., . . . Wilson, G. T. (2009). Mind the gap: Improving the dissemination of CBT. *Behav Res Ther, 47*(11), 902-909. doi:10.1016/j.brat.2009.07.003

Stice, E. (2002). Risk and maintenance factors for eating pathology: A meta-analytic review. *Psychological Bulletin, 128*(5), 825-848. doi:10.1037/0033-2909.128.5.825

Termorshuizen, J. D., Watson, H. J., Thornton, L. M., Borg, S., Flatt, R. E., MacDermod, C. M., . . . Bulik, C. M. (2020). Early impact of COVID-19 on individuals with self-reported eating disorders: A survey of ~1,000 individuals in the United States and the Netherlands. *The International journal of eating disorders, 53*(11), 1780-1790. doi:10.1002/eat.23353

van den Berg, E., Melisse, B., Koenders, J., de Jonge, M., Blankers, M., de Beurs, E., & Dekker, J. (2020). Online cognitive behavioral therapy enhanced for binge eating disorder: study protocol for a randomized controlled trial. *BMC Psychiatry, 20*(1), 190. doi:10.1186/s12888-020-02604-1

Versteegh, M. M., Vermeulen, K. M., Evers, S. M., de Wit, G. A., Prenger, R., & Stolk, E. A. J. V. i. h. (2016). Dutch tariff for the five-level version of EQ-5D. *19*(4), 343-352.

Waller, G., Pugh, M., Mulkens, S., Moore, E., Mountford, V. A., Carter, J., . . . Smit, V. (2020). Cognitive-behavioral therapy in the time of coronavirus: Clinician tips for working with eating disorders via telehealth when face-to-face meetings are not possible. *International Journal of eating disorders, 53*(7), 1132-1141. doi:10.1002/eat.23289

Wilson, G. T. (2011). Treatment of binge eating disorder. *The Psychiatric clinics of North America, 34*(4), 773-783. doi:10.1016/j.psc.2011.08.011

Zerwas, S. C., Watson, H. J., Hofmeier, S. M., Levine, M. D., Hamer, R. M., Crosby, R. D., . . . Bulik, C. M. (2017). CBT4BN: A Randomized Controlled Trial of Online Chat and Face-to-Face Group Therapy for Bulimia Nervosa. *Psychotherapy and Psychosomatics, 86*(1), 47-53.

APPENDIX A

Welkom bij de online geleide zelfhulpbehandeling voor eetstoornissen van Novarum genaamd 'Overwin je Eetbuien'.

Fijn dat je ervoor gekozen hebt deze behandeling te volgen, daarmee heb je jouw eerste stap gezet richting herstel!

Het programma van 'Overwin je eetbuien' is een geleide zelfhulpvariant op CBT-E. Dit programma heeft een reputatie opgebouwd als een toonaangevende behandeling voor mensen die eetbuiproblemen hebben. Voordelen van deze geleide zelfhulpbehandeling zijn de laagdrempeligheid, online vanuit je eigen omgeving en op de momenten dat het jou uitkomt werken aan jezelf en het overwinnen van je eetbuien, terwijl je daarnaast door een wekelijks telefonisch contact toch gebruik kunt maken van de ondersteuning en expertise van een in eetstoornissen gespecialiseerde behandelaar.

Deze geleide zelfhulpbehandeling duurt 12 weken. De module bestaat uit 8 stappen. Via internet lees je informatie en voer je opdrachten uit. Hierbij behorend dien je 2 keer per week een evaluatiesessie met jezelf te plannen om je voortgang te monitoren. Deze evaluatiesessies duren 15 tot 30 minuten. Het is belangrijk om ze van te voren in te plannen en jezelf niet toe te staan dat andere dingen voorrang krijgen. Daarnaast heb je wekelijks telefonisch contact met je behandelaar van Novarum. Ook zal je per mail het verzoek krijgen om online vragenlijsten (ROM metingen) in te vullen - om jouw klachten te monitoren en te kijken of het programma je helpt.

Voordat je met de eerste stap begint, is het belangrijk dat je deel 1 van het boek 'Overwin je eetbuien' hebt doorgenomen (in ieder geval de hoofdstukken 1, 4 en 5). Als je dit hebt gedaan, ben je klaar om te beginnen met Stap 1.

Wij wensen je veel succes bij het volgen van de behandeling!

Deze module is gebaseerd op het boek 'Overwin je eetbuien' van Christopher G. Fairburn.

Welkom!
We beginnen met een korte uitleg over deze online omgeving.

Wat ga ik doen?
Op deze beveiligde online behandelomgeving lees je informatie door en maak je oefeningen waardoor je weer grip op het eten zult krijgen. In totaal bestaat de eHealth uit 8 stappen en 12 weken.

Zie hier een overzicht van de stappen en het bijbehorende tempo:

Stap	Duur (totaal 12 weken)
Stap 1. Goed beginnen	1 week
Stap 2. Regelmatig eten	1 week
Stap 3. Alternatieven voor eetbuien	1 week
Stap 4. Probleemoplossing	1 week
Stap 5. De balans opmaken	1 week
Stap 6. Eetregelmodule<br>Stap 7. Lichaamsbeeldmodule	Samen 6 weken
Stap 8. Goed eindigen	1 week

Het is de bedoeling dat je per keer dat je aan de module werkt, een hele stap doorloopt. Let er op dat elke stap meerdere oefeningen betreft.

Elke week heb je telefonisch contact met je behandelaar. Hij/zij kan zien wat je ingevuld hebt en samen bespreken jullie hoe het gaat.

Waar vind ik mijn ingeleverde opdrachten?
Je vindt je afgeronde opdrachten terug in Mijn dossier. Daar kun je ze bekijken wanneer je maar wil en ook uitprinten.

Mijn planning
Geef aan op welke dagen jij de komende 12 weken jouw evaluatiesessies houdt en wanneer je de belcontacten hebt.

Doen

Dag

Tijdstip

Evaluatiesessie 1:

Evaluatiesessie 2:

Belcontact:

Hoe vul ik de eetregistratie in?
Doe je best om precies te zijn en dingen zo snel mogelijk nadat ze zijn gebeurd te noteren.

- **Kolom 1:** Noteer hier het tijdstip waarop je iets at of dronk.
- **Kolom 2:** Schrijf precies op wat je hebt gegeten of gedronken, met inbegrip van alles wat je tijdens een eetbui hebt genuttigd. Laat niets weg. Houd geen calorieën bij, maar geef een eenvoudige beschrijving van wat je hebt gegeten en gedronken. Doe dat zo snel mogelijk daarna, liefst meteen.

  Noteer in kolom 2 een accolade achter de producten die je gezamenlijk als 'hoofdmaaltijd' (ontbijt/lunch/avondeten) beschouwt door er een accolade achter te zetten. Dit is een accolade: }. Zet geen accolade achter tussendoortjes of andere eetepisodes. Zo kun je makkelijk het overzicht krijgen van welke hoofdmaaltijden je per dag heb gegeten.
- **Kolom 3:** Geef aan waar je was toen je het eten of drinken nuttigde. Als het thuis was, vermeld dan specifiek in welke kamer.
- **Kolom 4:** Noteer een sterretje in deze kolom bij het eten dat jij op dat moment als excessief ervoer of waarbij je controleverlies had.
- **Kolom 5:** Gebruik de B/L-kolom om bij te houden wanneer je braakt of laxeermiddelen of plaspillen misbruikt.
- **Kolom 6:** Gebruik deze kolom als een soort dagboek om alles te noteren wat invloed lijkt te hebben op je eetgedrag. Een voorbeeld: elke keer dat je een sterretje noteert in kolom 4, moet je in kolom 6 noteren wat de omstandigheden op dat moment waren. Dit heeft tot doel dat je voor elke episode van 'excessief eten' of 'controleverlies' kunt achterhalen wat de triggers waren. Misschien had je net ruzie met iemand gehad en was je boos. Of misschien stond je sociaal onder druk om te eten. Gebruik kolom 6 ook om iedere keer dat je jezelf weegt je gewicht te noteren.

Alternatieven voor eetbuien
Het aanwennen van een regelmatig eetpatroon houdt in essentie twee dingen in. Aan de ene kant het introduceren van de geplande maaltijden en tussendoortjes, aan de andere kant het niet eten in de tussenliggende periodes. Stap 3 helpt je om niet te eten in die tussenliggende periodes. Verder krijg je advies over hoe je kunt bepalen of je gewicht al dan niet verandert.

Je voorbereiden op het inzetten van alternatieve activiteiten
Wanneer je begint met het aanwennen van een regelmatig eetpatroon zal het vaker voorkomen dat je tussen de maaltijden en tussendoortjes door nog de drang voelt om te eten. En misschien ook wel de drang om na afloop te

braken. De gedachte dat deze drang net zolang oploopt totdat je je er niet meer tegen kunt verzetten, is een groot misverstand.

Wat gebeurt er dan wel? Je drang loopt op tot een piekhoogte en neemt daarna geleidelijk af. De uitdaging is om in de periode waarin de drang op zijn ergst is (van ongeveer een uur) hier niet aan toe te geven. Dit lukt het beste door jezelf actief af te leiden. Hoe je dit doet hangt van de omstandigheden af, maar het is handig om van tevoren een lijstje te maken met dingen die je zou kunnen doen.

G. De eerste stap

De eerste stap bestaat uit het bedenken van activiteiten die je kunnen helpen om weerstand te bieden aan de drang om te eten of te braken. Die activiteiten heb je nodig in de periode waarin de drang het meest intens is.

Hier zijn een paar voorbeelden van veel gekozen activiteiten:

- Een eind wandelen in pittig tempo
- Een stuk fietsen
- Vrienden of familieleden bellen of bezoeken
- Sporten
- E-mailen
- Facebooken
- Op internet surfen
- Gamen
- Een bad of douche nemen
- Een boeiende film of favoriete serie kijken

Mijn lijst van activiteiten

De bedoeling is dat je een lijst van activiteiten bedenkt die bij jou past.

Over het algemeen geldt voor elke activiteit dat deze aan drie kenmerken moet voldoen:

- Het is actief (je dóet iets) in plaats van passief (kijken naar wat toevallig op tv is).
- Het is plezierig (het voelt niet als verplicht).
- Het is realistisch (het is iets wat je waarschijnlijk echt zult doen).

Probleemoplossing

Oefenen

Beschrijf een probleem en bedenk oplossingen voor dit probleem. Kies vervolgens de beste oplossing uit, voer deze uit en evalueer de volgende dag hoe het hele proces is gegaan.

Je kunt met zo ongeveer elk soort probleem oefenen. Ook met problemen die helemaal losstaan van je eetprobleem, bijvoorbeeld problemen op het werk of privé.

Stap 1: Stel zo snel mogelijk vast wat het probleem is

Wanneer merkte je dat er zich mogelijk een probleem voor zou doen?

Stap 2: Omschrijf het probleem precies

Stap 3, 4 en 5: Oplossingen bedenken, tegen elkaar afwegen en een keuze maken

- Stap 3: Bedenk eerst zoveel mogelijk oplossingen

- Stap 4: Weeg de voor- en nadelen van elke oplossing tegen elkaar af
- Stap 5: Kies de beste oplossing of combinatie van oplossingen

Oplossing

Voor- en nadelen

Beste oplossing

- Ik kies deze oplossing
- Ik kies deze oplossing
- Ik kies deze oplossing
- Ik kies deze oplossing
- Ik kies deze oplossing

Stap 6: Handel in overeenstemming met de oplossing

Voer de oplossing uit. Je hoeft niet star aan de gekozen oplossing vast te houden. Als die toch niet zo'n goed idee blijkt te zijn, probeer je een van de andere oplossingen.

Stap 7: Evalueer je aanpak

Beschrijf hoe het hele proces om je probleem op te lossen is gegaan. Wat ging er goed en wat niet?

Je kan zo vaak oefenen met deze opdracht als je wil. Dit kun je doen met behulp van de opdracht 'Probleemoplossing' bovenin het scherm.

Klik op 'Vul in' om de opdracht te maken.

Eetregelmodule

Stap 1. Achterhaal welke voedingsmiddelen je vermijdt

De eerste stap is dan dat je achterhaalt welke voedingsmiddelen je vermijdt. De beste methode hiervoor lijkt misschien een beetje vreemd, maar werkt wel.

Ga naar een supermarkt in de buurt met een uitgebreid assortiment aan merken en soorten voedingsmiddelen en loop door alle gangpaden. Noteer in een notitieblok alle voedingsmiddelen die je liever niet zou eten. Dit kan zijn vanwege het effect dat jij denkt dat ze op je figuur of gewicht kunnen hebben of omdat je denkt dat het eten ervan een eetbui kan uitlokken.

Hieronder zie je een voorbeeld van zo'n lijst.

Stap 2. Verdeel de voedingsmiddelen in categorieën

Eenmaal thuis verdeel je jouw lijst (die vaak veertig of meer items bevat) in vier categorieën, gerangschikt naar de mate waarin je het moeite zou kosten om ze te gaan eten.

Categorie:

Het kost me weinig moeite

Voedingsmiddelen

Categorie:

Het kost me iets meer moeite

Voedingsmiddelen

Categorie:

Het kost me redelijk wat moeite

Voedingsmiddelen

Categorie:

Het kost me onvoorstelbaar veel moeite

Voedingsmiddelen

Stap 3. Introduceer de voedingsmiddelen in je dieet

Neem de voedingsmiddelen op in je geplande maaltijden en tussendoortjes. Doe dit alleen op dagen waarop je het gevoel hebt controle over je eetgedrag te hebben. Anders kunnen ze misschien een eetbui uitlokken.

Begin met etenswaren uit de gemakkelijkste categorie en houd dit een paar weken vol. Ga vervolgens door met de volgende categorie, en zo verder. Als het goed is, heb je binnen zes tot acht weken de meeste, zo niet alle, voedingsmiddelen in je dieet opgenomen.

Hoeveel je van een bepaal voedingsmiddel eet, maakt niet uit. Een beetje is al genoeg. Over het algemeen is het eerder de gedachte dat je dat voedingsmiddel hebt gegeten die een eetbui uitlokt dan de bezorgdheid over de gegeten hoeveelheid.

Voor sommige mensen is dit gemakkelijk, voor anderen minder. Hoe dan ook vergt het de nodige oefening.

Ga net zolang door met het introduceren van deze voedingsmiddelen tot je er geen moeite meer mee hebt. Het moment om ermee te stoppen is aangebroken als het eten van deze voedingsmiddelen je niet meer van je stuk brengt.

En onthoud

Als je geen voedingsmiddelen vermijdt, wordt je kans op eetbuien veel kleiner.

Het opvolgen van dit advies kan betekenen dat je dingen moet gaan eten die jij als dikmakend of ongezond beschouwt. Toch is het belangrijk om vol te houden. Eten dat dikmakend is, bestaat niet. Het hangt allemaal af van hoeveel je ervan eet.

Door deze voedingsmiddelen te introduceren, krijg je meer controle over je eetgedrag. Je neiging tot het hebben van eetbuien neemt namelijk af. En voor wat betreft het ongezonde ervan: je kunt deze voedingsmiddelen beter met mate eten dan er tijdens een eetbui naar te grijpen.

Bovendien hoef je deze voedingsmiddelen niet de rest van je leven te blijven eten. Als het eten ervan je niet langer angstig maakt, is het verstandig om wat rustiger aan te gaan doen met voedingsmiddelen die algemeen als ongezond worden beschouwd. Bijvoorbeeld dingen die rijk zijn aan verzadigde vetten of transvetten.

Probeer deze voedingsmiddelen niet volledig te ontlopen. Je kunt jezelf beter toestaan om ze af en toe te eten dan het jezelf te verbieden.

Tot slot: sommige mensen verklaren het feit dat ze een dieet volgen door te zeggen dat ze vegetarisch zijn of een voedselallergie hebben. Wat je reden voor een dieet ook is, elk dieet dat deels bedoeld is om af te vallen of je figuur te veranderen, is een vorm van lijnen. Als je te maken hebt met eetbuien, moet dit lijngedrag aangepakt worden.

Lichaamsbeeldmodule

Aan de slag

Vul de lichaamsbeeldregistratie in voor een werkdag en een vrije dag.

Doe je best om het op het moment zelf in te vullen en wees niet verbaasd of van streek als je veel moet opschrijven. Probeer gewoon alles vast te leggen, hoe verleidelijk het ook kan zijn om dingen weg te laten.

Uitleg

- Noteer het tijdstip.
- Noteer het elke keer dat jij je lichaam checkt of vermijdt, je bijzonder bewust wordt van je lichaam, je lichaam vergelijkt met dat van anderen, het lichaam van iemand anders inspecteert, of jezelf 'dik voelt'. Noteer op die momenten precies wat je deed. Zet tussen haakjes hoeveel minuten het gevoel of gedrag duurde.
- Noteer waar je op dat moment was.
- Noteer de context, gedachten en gevoelens die met het gedrag of de ervaring gepaard gingen.

Bekijk een ingevulde lichaamsbeeldregistratie

Gebruik van de spiegel

Je gebruik van de spiegel is het beste voorbeeld. Het zou niet goed of realistisch zijn om nooit in een spiegel te kijken. Vermijding is even problematisch als herhaaldelijk checken. In plaats daarvan moet het gedrag worden veranderd.

Hieronder staan de voornaamste vragen om aan jezelf te stellen:

Hoe vaak kijk ik per dag in de spiegel?

Maak een schatting
keer

Hoe lang kijk ik per keer?

Wat doe ik precies als ik in de spiegel kijk?

Wat probeer ik te weten te komen?

Kan ik dat op deze manier te weten komen?

Hoeveel verschillende spiegels gebruik ik thuis?

spiegels

Dit zijn vragen waarbij je misschien nog nooit hebt stilgestaan. Denk er goed over na. Als jij iemand bent die veel in de spiegel kijkt, waarom doe je dat dan? Wat hoop je te weten te komen? Veranderingen in je figuur zul je op deze manier niet ontdekken, tenzij je een fotografisch geheugen hebt. Daarvoor kun je beter op je gewichtsgrafiek afgaan.

Denk nu na over de onderstaande extra vragen, en lees de antwoorden door.

Wat zijn goede redenen om in de spiegel te kijken?

- Checken hoe je haar en kleding zitten.
- Vrouwen hebben een spiegel nodig om make-up aan te brengen en te verwijderen.
- Mannen hebben een spiegel nodig om zich te scheren.

Zijn er geen andere goede redenen om in de spiegel te kijken?
Nee. Spiegels zijn 'riskant' voor mensen met een eetprobleem. Er kan maar beter verstandig mee worden omgegaan.

Hoeveel spiegels kun je thuis het beste hebben?
Eén voor je gezicht en één voor je lichaam. De andere kun je het beste wegdoen, tenzij ze een puur decoratieve functie hebben. Het is moeilijk om overmatig gebruik van spiegels te vermijden als je een groot aantal spiegels om je heen hebt. Extra problematisch zijn spiegels in de badkamer.

Hoe kan ik de 'uitvergroting' voorkomen die het gevolg is van kritisch kijken?
Als je in de spiegel kijkt, zorg dan dat je je niet concentreert op bepaalde lichaamsdelen en vooral niet op delen waaraan je een hekel hebt. Kijk naar je lichaam als geheel, met inbegrip van de meer neutrale delen (bijv. handen, voeten, knieën, je haar).

Kijk verder ook naar de omgeving op de achtergrond, dit helpt namelijk om je een besef van verhoudingen te geven.

Hoe zit het met mezelf naakt in de spiegel bekijken?
Geen goed idee, tenzij je het doet om jezelf te bewonderen! Mensen met eetbuiproblemen richten hun blik echter eerder op lichaamsdelen waaraan ze een hekel hebben. Het lichaam wordt aan een kritisch onderzoek onderworpen.

Het is ook geen goed idee om je aan- of uit te kleden voor de spiegel.

Ik heb een spiegel nodig om te kiezen wat ik aan zal trekken, vooral als ik op stap ga
Misschien, maar sommige mensen besteden hier ontzettend veel tijd aan en proberen wel drie of meer outfits. Dit gaat meestal gepaard met een steeds groter wordende ontevredenheid over hun uiterlijk en een slinkend zelfvertrouwen. Als dit voor jou geldt, probeer je outfit dan te kiezen voordat je hem aantrekt (bijvoorbeeld door de kleding op je bed te leggen).

Je doel moet zijn om je bewust te worden van de manier waarop je de spiegel gebruikt
Probeer je bewust te zijn van de manier waarop je de spiegel gebruikt. Stel jezelf ook vragen over je spiegelgebruik voordat je in een spiegel kijkt.

Dit geldt ook voor de andere vormen van figuur checken.

Wat spiegels betreft, ga verstandig om met het gebruik ervan en probeer beter te worden in het interpreteren van wat je ziet.
