# Supplementary material for: Web-Based Guided Self-Help vs Treatment as Usual for Binge-Eating Disorder: A Randomized Clinical Trial
Source: JAMA Netw Open. 2025 Oct 10;8(10):e2536644. doi: 10.1001/jamanetworkopen.2025.36644 (PMC12514623; doi:10.1001/jamanetworkopen.2025.36644)
Supplement: Supplement 2. — eTable 1. Secondary Measures eMethods. Details of the Main Statistical Analyses eResults. eTable 2. Per-Protocol Analysis of Comparative Differences Between Groups per Assessment [file jamanetwopen-e2536644-s002.pdf]

Supplemental Online Content

van Beers E, Melisse B, Poelstra P, et al. Web-based–guided self-help vs treatment as usual for binge eating disorder: a randomized clinical trial. *JAMA Netw Open*. 2025;8(10):e2536644. doi:10.1001/jamanetworkopen.2025.36644

eTable 1. Secondary Measures
eMethods. Details of the Main Statistical Analyses
eResults.
eTable 2. Per-Protocol Analysis of Comparative Differences Between Groups per Assessment

This supplemental material has been provided by the authors to give readers additional information about their work.

© 2025 van Beers E et al. *JAMA Network Open*.

eTable 1. Secondary Measures

**Measurement tool**	**Measure**	**Timepoints**	**Sample internal consistency, α (items)**
**Eating Disorder Examination**[1]	Binge-free days<br>Global score<br>Abstinence from binge eating<br>Full remission*	End-of-treatment<br>20-weeks post-treatment	.86 (74)
**Eating disorder examination–questionnaire**[2]	Objective binge episodes in the previous 28 days<br>Global score	40 weeks post-treatment	.94 (21)
**Body mass index**	Participant weight in kilograms per metre squared (kg/m^2)	End-of-treatment<br>20-weeks post-treatment	N/A
**Working alliance inventory** [3,4]	Therapeutic alliance	End-of-treatment	.69 (36)
**Body shape questionnaire**[5]	Body shape dissatisfaction	End-of-treatment<br>20-weeks post-treatment	.97 (34)
**Clinical impairment assessment** [6]	Clinical impairment	End-of-treatment<br>20-weeks post-treatment	.95 (16)
**Dropout**	Treatment dropout	Ongoingly	N/A

* Full remission was defined as no binge eating episodes in the previous 28 days and an EDE score below clinical cutoff of 1.74-[1] (1 SD below United Kingdom community norms)

1. Fairburn CG, Cooper Z, O'Connor ME. Eating Disorder Examination. In: *Cognitive Behavior Therapy and Eating Disorders*. 16.0D. Guilford Press; 2008:265-308.

2. Fairburn CG, Beglin SJ. Eating Disorder Examination Questionnaire. In: *Cognitive Behavior Therapy and Eating Disorders*. 6.0. Guilford Pres; 2008:309-313.

3. Horvath AO, Greenberg LS. Development and validation of the Working Alliance Inventory. *J Couns Psychol*. 1989;36(2):223-233. doi:10.1037/0022-0167.36.2.223

4. Munder T, Wilmers F, Leonhart R, Linster HW, Barth J. Working alliance inventory-short revised (WAI-SR): Psychometric properties in outpatients and inpatients. *Clin Psychol Psychother*. 2010;17(3):231-239. doi:10.1002/CPP.658;WGROUP:STRING:PUBLICATION

5. Cooper PJ, Taylor MJ, Cooper Z, Fairburn CG. The development and validation of the body shape questionnaire. *International Journal of Eating Disorders*. 1987;6(4):485-494. doi:10.1002/1098-108x(198707)6:4<485::aid-eat2260060405>3.0.co;2-o

6. Bohn K, Fairburn CG. Clinical Impairment Assessment Questionnaire (CIA 3.0). In: *Cognitive Behavior Therapy and Eating Disorder*. Guilford Press; 2008.

eMethods. Details of the Main Statistical Analyses

To examine differences in outcomes between groups, linear mixed model analyses with restricted maximum likelihood estimation (continuous normally distributed measures), multilevel negative binomial regression (count variables) and multilevel binary logistic regression (dichotomous variables) were used. Normality of the distribution was tested by checking the normality of the residuals and the random effects. Potential count variables like number of binge episodes were tested for overdispersion. As these measures showed overdispersion, multilevel negative binomial regression was used instead of Poisson regression. The initial basic model was a two-level model with repeated measurements (level 1) nested within patients (level 2) with a two-way interaction testing the comparative differences between conditions over time (in days). First, various covariance structures for the repeated measures were compared (subsequently AR1, ARMA11, CS, ARH1, CSH) based on the -2 log likelihood and number of model parameters (with maximum likelihood estimation for the linear models). When the best fitting covariance structure was established, a random intercept was added. After that the possible addition of random slopes for time was tested (they were not added as they did not explain enough variance in any case). Subsequently, potential quadratic functions of time were compared with the linear model for the best model fit (comparing -2 log likelihood). The additional quadratic parameters were tested in the following sequence: first time-squared, and if that led to significant better fit based on -2 log likelihood, followed by time-squared and time-squared*group. For the final linear mixed models restricted maximum likelihood was used. Dependent variables in the linear mixed model analyses were: EDE global score, EDE-Q global score, BMI, *Clinical Impairment Assessment*, *Body Shape Questionnaire*, and *Working Alliance Inventory*. Dependent variables in the multilevel negative binomial regression were: EDE number of objective binge episodes, EDE-Q number of objective binge episodes. The dependent variables in the multilevel binary logistic regression were: EDE objective binge episodes none (0) versus at least one (>0) and EDE full remission (no binge-eating episodes in the previous 28 days and a global EDE score below the clinical cut-off of 1.74). Estimated marginal means were derived from the final multilevel models for all measures and were compared between groups. The non-inferiority procedure concerning the number of binge episodes started with a two-sided test of the estimated marginal means between groups at a specific measurement point (as was done for all outcome measures). Then the 95% confidence interval of the mean difference between groups of that test was used. An example; we found a non-significant difference between web based guided self-help and TAU for number of binge episodes at 20 weeks post treatment (1,27 vs 1,84; p=0.17). The mean difference was -0.58, CI -1.41 – 0.26. As +1 was not included in the interval, non-inferiority was concluded.

© 2025 van Beers E et al. *JAMA Network Open*.

eResults

Treatment Dropout and Care Consumption

Treatment dropout was defined as either failing to begin treatment or stopping treatment prematurely. No differences were found; dropout was 22.6% (21/93) for web-based guided self-help and 19.2% (18/94) for treatment-as-usual (χ21=0.33, p=.56). Of those who successfully completed treatment in the web-based guided self-help group, 85% (61/72) used all 12 sessions while 15% (11/72) used 10-11 sessions. Of participants who successfully completed treatment-as-usual, 70% (53/76) used all 20 sessions and 30% (23/76) used 14-19 sessions.

Therapist adherence

In the guided self-help group, 74.8% of sessions had maximum therapist adherence (5/5), while 3.8% of sessions scored either 1 or 2. In the treatment-as-usual group, 76.5% of sessions had maximum adherence, and 1.1% scored either 1 or 2.

Safety

Adverse events were evaluated in all participants who received at least one treatment session. In the web-based guided self-help group, one participant dropped out due to interfering trauma symptoms, for which they stopped treatment and were referred for trauma treatment. In the treatment-as-usual group, three adverse events occurred, one of which required treatment to stop. Of the two who continued, one participant with a history of headaches experienced the onset of severe headaches, while the other experienced trouble sleeping. The client who stopped treatment-as-usual experienced a psychiatric crisis due to comorbid attention-deficit and hyperreactivity (ADHD) and trauma symptoms, for which they referred for specialized care.

© 2025 van Beers E et al. *JAMA Network Open*.

eTable 2. Per-Protocol Analysis of Comparative Differences Between Groups per Assessment

**Assessment**	**Web-based guided self-help (n=93)**	**Treatment -as-usual (n=94)**	**Effect size**	**Contrast estimate (SE) [95% CI]**	***P* value**
**Objective binge episodes in the previous 28 days**					
End-of-treatment	3.02 [2.20 to 4.13]	3.94 [3.02 to 5.12]	-0.17	-0.92 (0.54) [-1.98 to 0.14]	.09
20-weeks post-treatment	1.27 [0.80 to 2.04]	1.88 [1.24 to 2.86]	-0.20	-0.61 (0.45) [-1.50 to 0.28]	.18
**Binge-free days**					
End-of-treatment	24.25 [22.98 to 25.59]	23.72 [22.68 to 24.82]	0.10	0.53 (0.65) [-0.75 to 1.80]	.42
20-weeks post-treatment	28.98 [26.83 to 31.31]	28.18 [26.30 to 30.19]	0.09	0.80 (1.35) [-1.85 to 3.46]	.55
**Global Score**					
End-of-treatment	1.27 [1.09 to 1.46]	1.26 [1.10 to 1.43]	0.01	0.01 (0.07) [-0.14 to 0.16]	.91
20-weeks post-treatment	1.53 1.31 to 1.75]	1.51 [1.31 to 1.72]	0.02	0.01 (0.13) [-0.23 to 0.26]	.91
**Abstinence from binge eating (%)**					
End-of-treatment	63.39 [49.38 to 75.45]	73.35 [61.94 to 82.31]	-	-9.96 (7.69) [-25.06 to 5.14]	.20
20-weeks post-treatment	60.70 [47.03 to 72.87]	54.09 [41.55 to 66.14]	-	6.60 (8.12) [-9.35 to 22.55]	.42
**Full remission (%)**					
End-of-treatment	51.09 [37.24 to 64.78]	60.61 [48.22 to 71.78]	-	-9.52 (8.23) [-25.69 to 6.65]	.25
20-weeks post-treatment	44.04 [30.89 to 59.09]	43.47 [31.59 to 56.15]	-	0.58 (8.37) [-15.86 to 17.01]	.95
**Eating disorder examination–questionnaire, 40 week post-treatment**					
Objective binge episodes in the past 28 days	2.40 [1.64 to 3.51]	2.26 [1.59 to 3.22]	0.04	0.13 (0.56) [-0.96 to 1.23]	.81
Global score	2.10 [1.85 to 2.34]	1.76 [1.54 to 1.99]	0.33	0.34 (0.14) [0.06 to 0.61]	.02
**Body mass index (kg/m²)**					
End-of-treatment	33.50 [32.68 to 34.31]	34.96 [34.27 to 35.66]	-0.44	-1.47 (0.41) [-2.28 to -0.65]	<.001
20-weeks post-treatment	33.36 [32.19 to 34.53]	35.26 [34.19 to 36.33]	-0.39	-1.90 (0.72) [-3.32 to -0.47]	.01
**Working alliance inventory**					

© 2025 van Beers E et al. *JAMA Network Open*.

End-of-treatment	158.69 [154.23 to 163.16]	161.88 [157.90 to 165.85]	-0.17	-3.19 (2.57) [-8.24 to 1.87]	.22
**Body shape questionnaire**					
End-of-treatment	93.12 [88.07 to 98.17]	94.35 [89.85 to 98.84]	-0.06	-1.23 (2.22) [-5.60 to 3.14]	.58
20-weeks post-treatment	84.06 [78.72 to 89.40]	80.48 [75.68 to 85.27]	0.16	3.59 (2.60) [-1.52 to 8.69]	.17
**Clinical impairment assessment**					
End-of-treatment	13.58 [12.04 to 15.02]	13.35 [11.97 to 14.72]	0.04	0.23 (0.68) [-1.10 to 1.57]	.73
20-weeks post-treatment	11.08 [9.45 to 12.71]	9.42 [7.96 to 10.89]	0.25	1.66 (0.79) [0.10 to 3.22]	.04

© 2025 van Beers E et al. *JAMA Network Open*.
