# Supplementary material for: Web-Based Guided Self-Help vs Treatment as Usual for Binge-Eating Disorder: A Randomized Clinical Trial
Source: JAMA Netw Open. 2025 Oct 10;8(10):e2536644. doi: 10.1001/jamanetworkopen.2025.36644 (PMC12514623; doi:10.1001/jamanetworkopen.2025.36644)
Supplement: Supplement 3. — Data Sharing Statement [file jamanetwopen-e2536644-s003.pdf]

Data Sharing Statement

van Beers. Web-Based Guided Self-Help vs Treatment as Usual for Binge Eating Disorder. *JAMA Netw Open*. Published October 10, 2025. doi:10.1001/jamanetworkopen.2025.36644

Data
Additional Information: CCMO Overview of Medical Research in the Netherlands, NL76368.100.21.
Data available: Yes
Data types: Deidentified participant data
How to access data: Data will be made available upon reasonable request from the corresponding author.
When available: With publication

Supporting Documents
Document types: None

Additional Information
Who can access the data: The data will be made available to researchers whose proposed use of the data has been approved.
Types of analyses: The data will be made available for research purposes.
Mechanisms of data availability: The data will be made available with a signed access agreement and limited investigator support.
